# Germline determinants of aberrant signaling pathways in cancer

**DOI:** 10.1038/s41698-024-00546-5

**Published:** 2024-03-01

**Authors:** Davide Dalfovo, Riccardo Scandino, Marta Paoli, Samuel Valentini, Alessandro Romanel

**Affiliations:** Department of Cellular, Computational and Integrative Biology (CIBIO), University of Trento, 38123 Trento, (TN) Italy

**Keywords:** Computational biology and bioinformatics, Cancer genetics, Cancer genomics, Biomarkers

## Abstract

Cancer is a complex disease influenced by a heterogeneous landscape of both germline genetic variants and somatic aberrations. While there is growing evidence suggesting an interplay between germline and somatic variants, and a substantial number of somatic aberrations in specific pathways are now recognized as hallmarks in many well-known forms of cancer, the interaction landscape between germline variants and the aberration of those pathways in cancer remains largely unexplored. Utilizing over 8500 human samples across 33 cancer types characterized by TCGA and considering binary traits defined using a large collection of somatic aberration profiles across ten well-known oncogenic signaling pathways, we conducted a series of GWAS and identified genome-wide and suggestive associations involving 276 SNPs. Among these, 94 SNPs revealed *cis*-eQTL links with cancer-related genes or with genes functionally correlated with the corresponding traits’ oncogenic pathways. GWAS summary statistics for all tested traits were then used to construct a set of polygenic scores employing a customized computational strategy. Polygenic scores for 24 traits demonstrated significant performance and were validated using data from PCAWG and CCLE datasets. These scores showed prognostic value for clinical variables and exhibited significant effectiveness in classifying patients into specific cancer subtypes or stratifying patients with cancer-specific aggressive phenotypes. Overall, we demonstrate that germline genetics can describe patients’ genetic liability to develop specific cancer molecular and clinical profiles.

## Introduction

Common germline variants in the form of Single Nucleotide Polymorphisms (SNPs) represent the main form of DNA polymorphism. In the last fifteen years, genome-wide association studies (GWAS) identified thousands of variants linked with susceptibility to different types of cancers^1–3^. However, most of these variants exhibited low relative risk, suggesting that they individually have a small effect on the heritability of cancer^4–6^. Polygenic scores hence emerged as an effective approach to integrate multiple small effects across hundreds or even thousands of variants summarizing in a single measure the patients’ genetic liability to develop specific cancer types^7^.

Cancer, however, is a complex disease^8^ influenced by both germline variants and a heterogeneous landscape of somatic aberrations acquired during tumor formation and evolution which recurrently target core cellular pathways and processes^9^. A growing number of studies support the presence of intricate links between germline variants and somatic aberrations. For example, a pan-cancer study^10^ exploiting genomic data for >5000 tumors revealed hundreds of significant associations between germline variants and tumor formation in specific tissues or somatic aberration of specific cancer genes. Further, in^11^ a network-based approach was developed to study interactions between multiple germline variants and acquired somatic events in breast cancer, and in^12^ we queried genomic data from more than 500 prostate cancer patients and found strong signal of association between a germline SNP and SPOP mutated prostate cancer molecular subtype. In addition, in^13^ it was demonstrated that germline variants regulate the expression of cancer genes and associate both with local and global somatic mutations, and in^14^ it was recently demonstrated that polygenic background underlying common hematological traits influence the clonal selection of specific somatic mutations and the development of specific hematological cancer subtypes.

Overall, although there is an increasing evidence suggesting an interplay between germline and somatic variants and a large number of somatic aberrations in specific pathways are now used as hallmarks in many well-known forms of cancer^15^, an exhaustive exploration of the interaction landscape between germline variants and the aberration of these pathways in cancer is still largely missing.

Here we exploit data from The Cancer Genome Atlas (TCGA)^16^, ICGC Pan-Cancer Analysis of Whole Genomes (PCAWG)^17^ and Cancer Cell Line Encyclopedia (CCLE)^18,19^ projects, together with other cancer-specific studies, to integrate germline genotypes with somatic aberration profiles in a set of well-characterized oncogenic signaling pathways to obtain a pan-cancer and cancer-specific view of how common germline SNPs may contribute or predispose to the progression and evolution of tumors. We first identify and characterize an array of common SNPs that increase or decrease the predisposition of these somatic events patterns to occur and then exploit the theory of polygenic scores to explore to what extent germline genetics correlates with somatic molecular profiles, tumor subtypes, and clinical variables such as patients’ survival and tumor aggressiveness.

## Results

### SNP genotypes associate with somatic aberrations in oncogenic signaling pathways

**Fig. 1:**
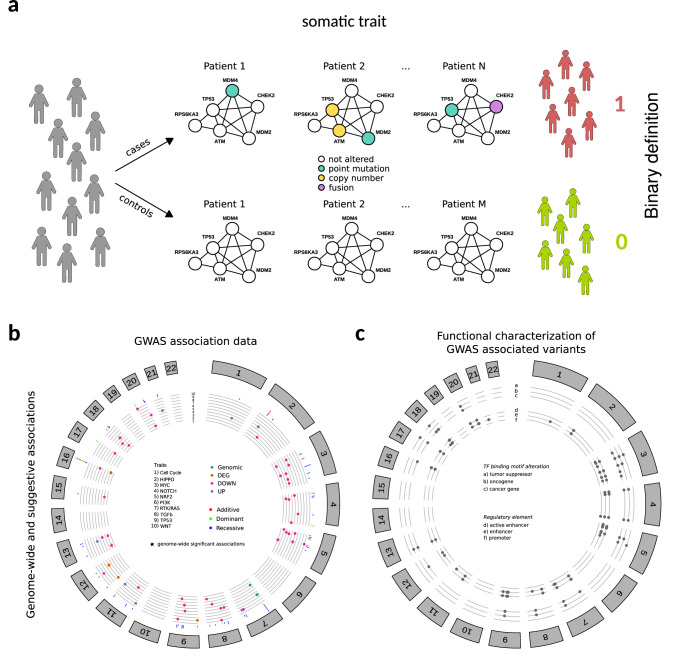
Somatic trait definition and GWAS analysis results. **a** Cancer patients are stratified based on the presence of aberrant genes in specific oncogenic signaling pathways to build binary somatic traits. TP53 somatic trait construction is shown as example. **b** Circular plots showing GWAS results for genome-wide significant associations (highlighted with the star symbol) and suggestive associations with *p*-value < 1e-07. The chromosomal positions (outer track) of the associations are shown for the forty traits in the inner track. The associations for different oncogenic pathways are reported on different rows and shown with different colors based on the trait’s definition. In the middle track, the statistical models used for each association are shown in different colors. **c** Circular plots showing functional characterization of genome-wide significant associations (highlighted with the star symbol) and suggestive associations with *p*-value < 1e-07. The functional characterization is performed on LD extended associated variants. LD extended sets of associated variants are characterized for genomic overlaps with regulatory elements (inner track) and to cause a change in the transcription factor binding motifs of genes implicated in cancer (middle track). The chromosomal positions (outer track) are reported for the corresponding variant from the GWAS analyses.

To examine to what extent germline genetics primes aberrations in oncogenic signaling pathways we first conducted genome-wide association studies (GWAS) using >8500 human samples across 33 cancer types characterized by TCGA and exploiting phenotypic traits built considering 10 oncogenic signaling pathways previously described and characterized in^20^; considered pathway include Cell Cycle, HIPPO, MYC, NOTCH, NRF2, PI3K, RTK RAS, TGF Beta, TP53 and WNT. Specifically, using TCGA SNP Affymetrix 6.0 array data, a collection of pan-cancer GWAS were performed by means of logistic regression considering the genotypes of 833,130 high-quality SNPs across 8682 TCGA high-quality normal samples (patient’s control samples, non-tumor) using additive, dominant and recessive models. Forty binary traits were tested, 10 of which considering for each oncogenic signaling pathway the presence/absence of a somatically altered gene (as described in^20^ and here referred to as *somatic traits*, Fig. 1a*)*, and the remaining ones (here referred to as *somatic transcriptomic traits*, Supplementary Fig. 1a) considering for each pathway the presence/absence of up-regulated genes (10 traits), down-regulated genes (10 traits) or generally deregulated genes (10 traits). The aberration frequencies of all traits across all tumor types are reported in Supplementary Fig. 2. All analyses were adjusted for age at diagnosis, sex, and the first six components from a principal component analysis (Supplementary Fig. 3). Genomic inflation (GI) was inspected (Supplementary Fig. 4) and TP53 downregulation recessive trait (TP53 DOWN recessive) was removed due to an inflation >1.1. In addition, heterogeneity of associations across tumor types was determined and investigated.

We identified 6 genome-wide significant (*p*-value < 4.2e-10) associations between 6 SNPs (1 intronic and 5 intergenic) and 5 traits (Fig. 1b, Supplementary Data 1), no one reported in the GWAS catalog^21^ or listed in^10^. We also identified additional 320 suggestive (*p*-value < 1e-06) associations between 272 SNPs (3 exonic, 7 promoter, 2 3′UTR, 85 intronic and 175 intergenic) and 36 traits, 7 already reported in the GWAS catalog, one associated with *Core binding factor acute myeloid leukemia* and six associated to non-cancer traits **(**Fig. 1b, Supplementary Fig. 1b, Supplementary Data 1), and no one listed in^10^. Of these suggestive associations, 8 had a *p*-value < 1e-08 and 71 a *p*-value < 1e-07. Overall, the majority of associations were trait-specific, with 39 SNPs associated to at least two traits. We found both risk and protective alleles with associations, especially those derived from dominant and recessive models, often exhibiting high/low ORs. In particular, recessive models applied in the association of low frequency variants and low case/control ratios resulted in significant though unstable results (high ORs and large CIs), demanding for careful interpretation of effect sizes. Of all 326 associations, about 97% demonstrated zero to moderate heterogeneity across tumor types (64% of associations with ${I}^{2}=0$, 21% with $0 < {I}^{2} < 0.25$ and 13% with $0.25\le {I}^{2} < 0.5$) while of the remaining ones only 1 had ${I}^{2}\ge 0.75$. All 9 associations with ${I}^{2}\ge 0.5$ were recessive, suggesting that the variable sample size of the different tumor type datasets (from 36 in the CHOL and DLBC datasets to 953 in the BRCA dataset) was probably the major contributor^22^ for the high heterogeneity of those associations. Of note, the global Minor Allele Frequency (MAF) distribution of genome-wide significant SNPs was not significantly different than the MAF distribution of suggestive SNPs (Supplementary Fig. 5). Linkage disequilibrium (LD) analysis was performed to retrieve variants in strong LD (D’ = 1 and *R*^2^ ≥ 0.8) with associated SNPs, obtaining 1105 LD variants for 133 associated SNPs.

Using our resource CONREL^23^ we found that 654 of the LD extended associated SNPs (59%) lie in enhancer elements conserved across 34 tissue types, 331 SNPs (30%) lie in active enhancer elements conserved across 33 tissue types and 15 SNPs lie in promoter regions (Fig. 1c, Supplementary Fig. 1c and Supplementary Data 2). Exploiting our resource Polympact^24^ we found that 523 of the 678 functional SNPs we identified (77%) cause a putative absolute relative change >0.5 in the scores of 594 transcription factor binding motifs, of which 19 are oncogenes (including *MYC*, *JUN*, and *CTNNB1*), ten are tumor suppressor genes (including, *TP53, PTEN, BRCA1,* and *CEBPA*) and more generally 90 (15%) are genes implicated in cancer (Fig. 1c, Supplementary Fig. 1c and Supplementary Data 2).

Overall, the data support the presence of wide association signal between functional germline SNPs and the occurrence of somatic aberrations in specific oncogenic signaling pathways.

### Associated variants are functionally linked to oncogenic signaling pathways

To further explore GWAS results, we asked whether the observed associations could be due to downstream effects that SNPs may have on the transcription of genes linked to the activity of traits’ oncogenic signaling pathways. We hence exploited *cis*-eQTL and transcriptomic data available from the Genotype-Tissue Expression (GTEx) project^25^ to search, among the 276 GWAS-associated variants, for *cis* interactions with genes in the pathways, or *cis* interactions with genes co-expressed and functionally close to genes in the pathways.

**Fig. 2:**
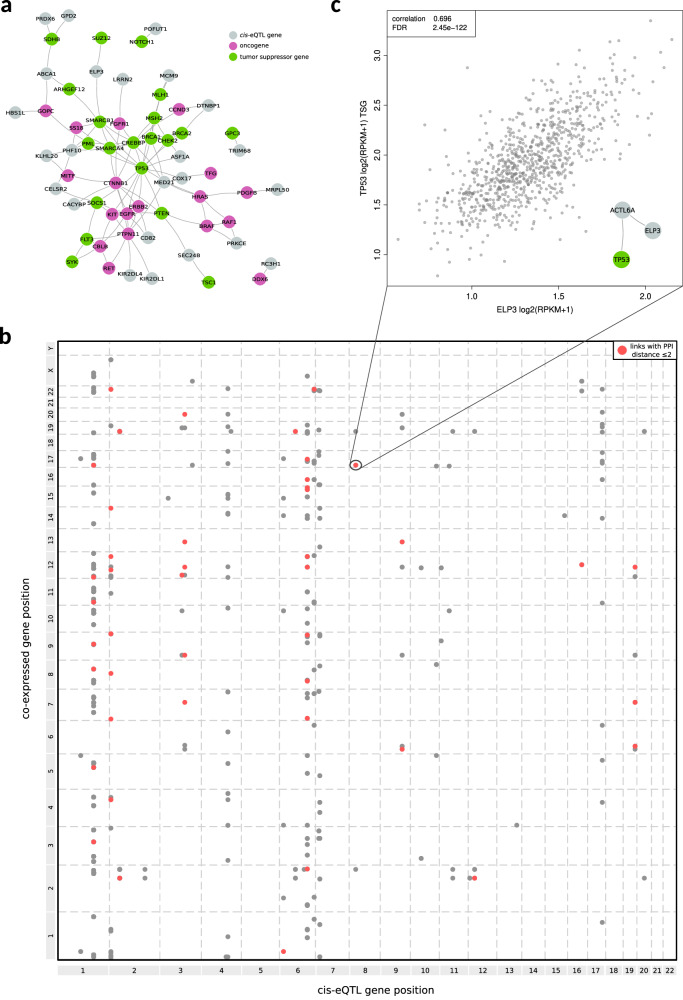
*cis*-eQTL and co-expression analyses. **a** PPI network showing *cis*-eQTL genes that were found connected to cancer-related genes. **b** Grid visualization highlighting coordinates of *cis*-eQTL genes in one dimension and coordinates of co-expressed genes in the other dimension. Points in red represent links between genes with PPI interaction data supporting a close link (PPI distance ≤2) between the two proteins. **c** An example representing variant rs2722888 alternative allele (associated with TP53 somatic trait) linked to increased expression of *ELP3* gene in *Whole Blood* tissue, which was positively correlated with *TP53* transcript level with PPI interaction data supporting a close link (PPI distance 2) between the two proteins.

Overall, we retrieved 247 *cis*-eQTL links (of which 123 identified across multiple GTEx tissues) involving 94 variants and 134 transcripts (Supplementary Data 3). Of these transcripts, 89 were protein-coding genes with an associated gene symbol, while the remaining ones were mostly categorized as novel transcripts. Interestingly, although only three of these 89 *cis*-eQTL genes are known to be involved in cancer, when exploiting data from an integrated protein-protein interaction (PPI) network 66% of the 74 *cis*-eQTL genes that are characterized in the PPI network were found connected to genes involved in cancer, of which 15 were connected to oncogenes and 16 were connected to tumor suppressor genes (Fig. 2a). Further, of the 89 *cis*-eQTL genes 53 demonstrated significant transcript level correlations with oncogenic signaling pathway-related genes, 25 of which exhibiting consistent significant correlations across multiple tissues (Supplementary Data 4). Of note, those co-expression signals span across several traits, with some oncogenic pathways exhibiting enriched signal in specific traits, like downregulation-based somatic transcriptomic traits, which show the richest signal.

Overall, 50 SNPs were involved in *cis* interactions with genes that were observed co-expressed with members of the corresponding traits’ oncogenic pathways, for a total of 1802 putative links (Fig. 2b and Supplementary Data 4). Interestingly, mean PPI distance among *cis*-eQTL genes and co-expressed genes was 2.94, a distance that was smaller (*p*-value < 1e-03) when compared to the ones obtained from permuted gene sets. Of note, 205 putative links demonstrated a distance less than or equal to 2. Among those latter links, we may highlight variant rs2722888, a SNP we found associated to TP53 somatic trait (additive), which was observed with an effect size lower than 1 (Supplementary Data 1). This indicates that aberrations in TP53 pathway is less likely to occur when the alternative allele is present. Interestingly, variant rs2722888 alternative allele was linked to increased expression of *ELP3* gene in multiple GTEx tissues, which was positively correlated (correlations across tissues in the range 0.6–0.7) with *TP53* transcript level (Fig. 2c, Supplementary Fig. 6a and Supplementary Data 4) with PPI interaction data supporting a close link (PPI distance 2) between the two proteins. We can hence speculate that patients carrying rs2722888 SNP may constitutively have higher expression of *TP53* gene, likely protecting cells from the accumulation of somatic aberrations in the TP53 signaling pathway and hence supporting the observed GWAS association.

Another interesting example is variant rs12686004, which was found additively associated to Cell Cycle downregulation trait (Cell Cycle DOWN additive) with an OR of 3.4 (Supplementary Data 1), indicating a strong enrichment of variant’s alternative allele in patients with downregulation of genes part of the Cell Cycle pathway. Variant rs12686004 alternative allele was linked to increased expression of *ABCA1* gene, which was negatively correlated (−0.7) with *RB1* transcript level (Supplementary Fig. 6b and Supplementary Data 4) and closely linked (PPI distance 2) to it. Interestingly, *RB1* is a tumor suppressor gene and is dysfunctional in many major cancers^26^. Hence, we can hypothesize that patients carrying rs12686004 SNP may constitutively have lower expression of *RB1* gene, likely enhancing the cancerous phenotype of cells that accumulate a somatic deregulation of Cell Cycle genes.

Further, we may highlight variant rs436898, associated with NRF2 downregulation trait (NRF2 DOWN recessive). The SNP was found linked to increased expression of *TMEM30A* gene in multiple GTEx tissues, which was in turn negatively correlated to *KEAP1* gene expression (correlations across tissues in the range 0.53–0.58) and closely PPI connected to it (Supplementary Fig. 6c, d and Supplementary Data 4). Based on these observations, GWAS association of rs436898 variant can be supported by the observation that patients carrying the SNP may have reduced expression of *KEAP1*, which combined with somatic downregulation of other NRF2 pathway genes likely exposes cells to a cancerous phenotype characterized by an increased induction of NRF2.

Taken together, these results support the hypothesis that functional links between GWAS-associated variants, the corresponding traits’ oncogenic signaling pathways and cancer genes exists, further strengthening the validity of our GWAS results.

### Polygenic somatic scores

Provided the strong and broad association signal we identified in the TCGA dataset and the putative functional links we observed, we then explored to what extent polygenic scores can capture the relationship between the unique combination of alleles in a cancer patient and its likelihood to present aberrations in specific oncogenic signaling pathways. A new class of polygenic scores, referred to as *Polygenic Somatic Scores (PSS)*, were computed in the TCGA dataset for all considered traits across additive, recessive, and dominant models using a five-fold cross-validation approach. Given a trait, the computational strategy we developed first identifies the best *p*-value cutoff to build the PSS across different LD clumps, then determines the PSS performances in terms of AUC across the different LD clumps, selecting the best performing one, and finally determines its statistical significance using permutation analysis and multiple hypotheses correction.

**Fig. 3:**
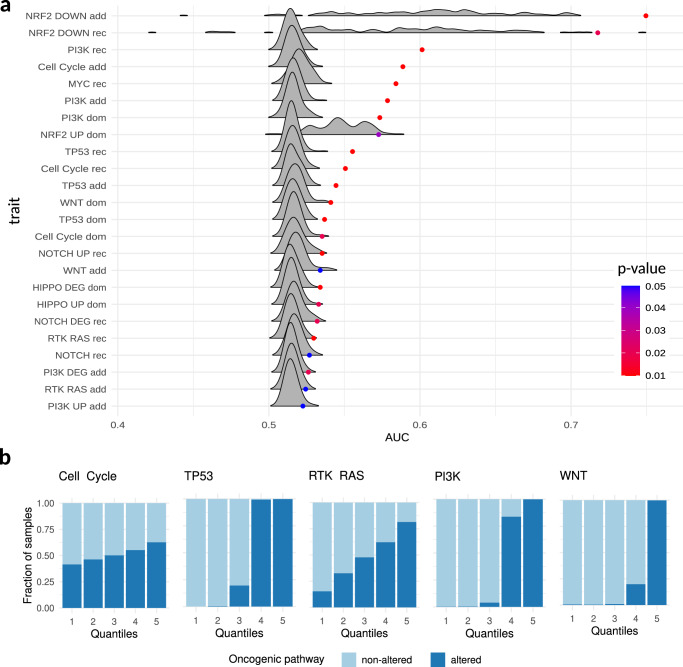
Polygenic somatic score (PSS) analysis. **a** Ridgeline plot of all PSS with a FDR smaller than 0.25, ordered by AUC value, showing the distribution of AUC values generated from random permutations and the observed AUC values (dots) colored by the corresponding *p*-value. **b** Quantile plots with 5 quantiles of increasing PSS for all the somatic traits with significant FDR using the additive model showing the fraction of samples with altered and non-altered phenotypes.

Overall, we observed 24 PSS showing an FDR < 0.25 across 9 oncogenic signaling pathways and different association models (Supplementary Data 5). Among the obtained PSS, NRF2 downregulation traits (NRF2 DOWN) presented consistent high AUC values across the different association models with an AUC of 0.75 for the additive model and 0.72 for the recessive model. Of note, the baseline distributions built on NRF2 transcriptomic traits show a high variance due to the low ratio between cases and controls patients (0.3% for NRF2 DOWN and 1.6% for NRF2 UP). The other somatic traits, including traits for Cell Cycle, TP53, MYC, PI3K, and RTK RAS oncogenic pathways were observed with AUC values ranging from 0.53 to 0.61 and with an observed AUC greater than all the corresponding baseline distribution values (Fig. 3a). As shown in Fig. 3b, quantile plots obtained from PSS calculated using the identified LD clump and *p*-value thresholds but exploiting the entire TCGA dataset clearly demonstrate how high PSS predominantly identify patients with altered oncogenic pathways. As shown in Supplementary Fig. 7, no specific tumor type is segregated by our PSS.

The 24 PSS with FDR < 0.25 (Fig. 3a), denoted as *pan-cancer PSS (pPSS)*, were retained for further analyses.

### PSS associate with patient’s clinical endpoints

**Fig. 4:**
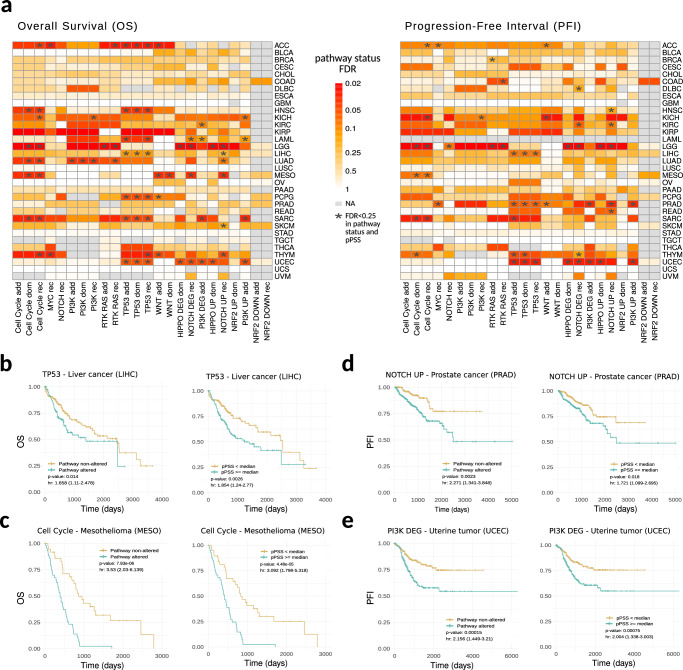
Clinical endpoints analysis. **a** Tile plots recapitulating the traits survival analysis results. Results are divided based on PFI and OS events. For each trait’s oncogenic pathway aberrations status and tumor type, corrected (FDR) empirical *p*-values computed comparing the observed AUC with the corresponding AUC baseline reference distribution are reported. Combinations of trait and tumor type were both trait’s pathways aberration status and pPSS survival analyses resulted statistically significant (FDR < 0.25) are highlighted with an asterisk. **b**–**e** Kaplan–Meier curves showing significant survival analyses for specific examples in both trait’s pathway aberration status (left) and pPSS (right).

To determine the effectiveness of pPSS, we first explored to what extent they can reproduce the prognostic value of somatic (transcriptomic) traits. Tumor types were analyzed separately and Overall Survival (OS) and Progression-Free Interval (PFI) data for TCGA patients was retrieved from^27^. Patients were stratified based on both traits’ oncogenic pathways aberration status and pPSS quantiles (considering the median values) and tumor type-specific analyses were performed using a Cox proportional hazards regression model considering age, sex, and principal components as covariates. Also in this case, models’ performances (AUC) were computed using a five-fold cross-validation approach and were then tested for statistical significance against reference baseline distributions generated using permutation analyses, finally correcting for multiple hypotheses. Overall, we observed 87 significant (FDR < 0.25) traits showing also a significant (FDR < 0.25) pPPS (70 from OS analysis, 46 from PFI analysis) across 19 tumor types (Fig. 4a, Supplementary Data 6). pPSS reproduced traits’ OS and PFI prognostic value across different tumor types, with Cell Cycle and TP53 somatic traits showing significant OS associations across 8 tumor types and significant PFI associations across 6 and 5 different tumor types, respectively. As examples, TP53 pathway aberrations status and pPSS (TP53 additive trait) showed a strong OS prognostic value in LIHC tumors (Fig. 4b), Cell Cycle pathway aberrations status and pPSS (Cell Cycle dominant trait) demonstrated OS prognostic value in MESO tumor (Fig. 4c), NOTCH UP pathway aberrations status and pPSS (NOTCH UP recessive trait) demonstrated PFI prognostic value in PRAD (Fig. 4d) and PI3K DEG pathway aberrations status and pPSS (PI3K DEG additive trait) showed significant PFI prognostic value in UCEC tumors (Fig. 4e).

Overall, our data demonstrate that pPSS can be potentially used to stratify patients with poor survival or treatment response.

### PSS and tumor subtypes

**Fig. 5:**
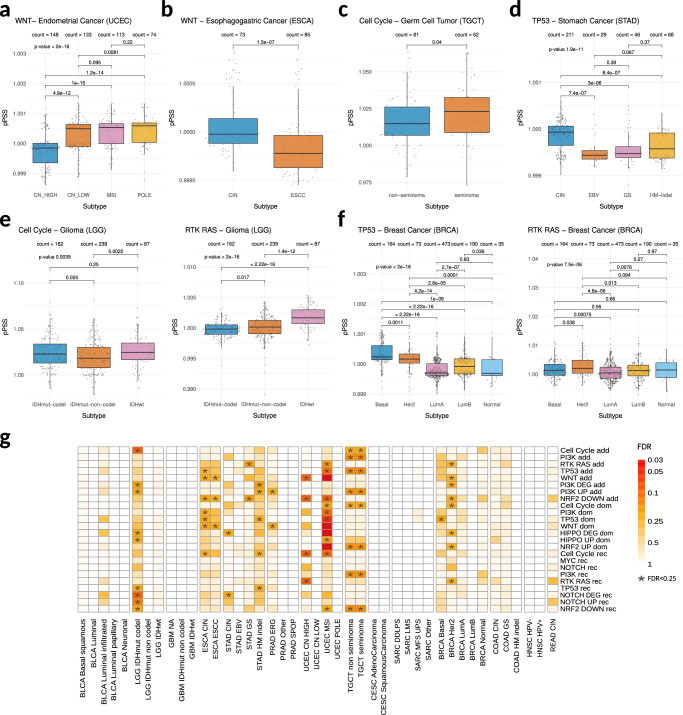
pPSS and tumor subtypes. **a**–**f** Boxplots showing the distributions of the pPSS values across different tumor subtypes. pPSS in each cancer subtype are compared using Kruskal–Wallis test and pPSS for each cancer subtypes pair are compared using Wilcoxon-test. **g** Tile plot recapitulating the tumor subtype analysis results. For each pPSS and tumor subtype, FDR values of empirical *p*-values computed comparing the observed AUC with the corresponding baseline reference distribution are reported. The combinations of pPSS and tumor subtype statistically significant (FDR < 0.25) are highlighted with ‘*’.

We then asked to what extent pPSS can be used to identify tumor-specific subtypes. For each tumor type, we tested the presence of a significant deviation in the distribution of pPSS across different tumor subtypes. Interestingly, we identified several tumor types were pPSS demonstrated strong shifts across specific subtypes (Fig. 5). Examples are UCEC *CN_HIGH* subtype (Fig. 5a), ESCA *CIN* subtype (Fig. 5b), TGCT *non-seminoma* and *seminoma* subtypes (Fig. 5c), STAD *CIN* subtype (Fig. 5d), LGG *IDHmut codel* subtype (Fig. 5e), BRCA *Basal* and *Her2* subtypes (Fig. 5f). Of note, several pPSS demonstrated significant shifts across subtypes of multiple tumor types.

To explore further this relationship, we built logistic regression models and by comparing observed AUC against AUC baseline distributions obtained from permutation analysis, we identified 22 pPSS across the subtypes of 7 tumor types with statistically significant (FDR < 0.25) classification performances (Fig. 5g, Supplementary Data 7). Additionally, in most of those cases an extended logistic regression model integrating all significant subtype-specific pPSS achieved same or better performances in classifying tumor subtypes (Supplementary Data 8). In particular, integrated models for subtypes UCEC *CN_HIGH*, TGCT *non-seminoma* and TGCT *seminoma* achieved much better classification performances with respect to models built with single pPSS. Instead, integrated models for subtypes BRCA *Basal*, BRCA *Her2*, STAD *CIN*, STAD *GS*, ESCA *CIN,* and ESCA *ESCC* exhibited classification performances that were comparable to the single most significant pPSS. Of note, the majority of the subtype-specific pPSS were non-transcriptomic and combinations of Cell Cycle, NRF2 DOWN, PI3K, TP53, and WNT pPSS were observed as particularly effective in identifying specific tumor subtypes.

Overall, our results demonstrate that pPSS can be used across several tumor types to stratify patients based on specific tumor subtypes.

### Validation of PSS in an independent pan-cancer dataset

We next tested the effectiveness of our 15 non-transcriptomic pPSS using data from the ICGC PCAWG project^17^, a large collection of cancer and matched normal whole genomes from patients spanning over 40 tumor types. Although the differences in PCAWG and TCGA projects data collection limit our ability to test and validate pPSS in PCAWG patients, we exploited PCAWG germline and somatic processed data to test the presence of statistically significant shifts in the distribution of pPSS among PCAWG patients with somatic trait-specific aberrations.

**Fig. 6:**
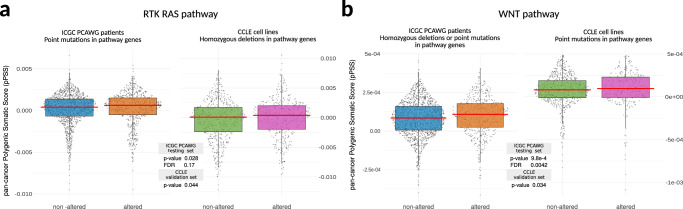
pPSS validation using data for ICGC PCAWG and CCLE. Boxplots showing statistically significant shift of pPSS distributions in patients harboring specific aberrations in somatic traits. Specific examples for RTK RAS (**a**) and WNT (**b**) pathways significant in ICGC PCAWG dataset (left) and confirmed in the CCLE dataset (right) are reported. Wilcoxon-test was performed (two-tail statistic with FDR correction for ICGC PCAWG and one-tail statistic for further confirmation in CCLE) and reported in the figure.

In detail, by exploiting GWAS summary statistics trained in the TCGA dataset, PCAWG germline genotype calls were used to calculate the 15 pPSS of interest across 1823 PCAWG patients. Somatic trait-specific aberrations for each patient were determined considering (separately or in combination) reported somatic point mutations, homozygous deletions, and amplifications data identified within the corresponding oncogenic signaling pathways. For 5 of the 15 tested pPSS (33%) we found a statistically significant (FDR < 0.25) increase of pPSS distribution in PCAWG patients harboring somatic trait-specific aberrations (Supplementary Data 9). For example, patients harboring point mutations in RTK RAS signaling pathway genes showed increased RTK RAS pPSS values (Fig. 6a, left) and patients harboring homozygous deletions or point mutations in WNT signaling pathway genes showed increased WNT pPSS value (Fig. 6b, left).

Overall, the predictive power of pPSS in identifying patients’ genetic liability to develop specific cancer molecular profiles was validated in an independent pan-cancer dataset.

### Validation of PSS in cancer cell line data

The 5 pPSS showing significant associations in the ICGC dataset were further tested for confirmation using data from the Cancer Cell Line Encyclopedia CCLE^18,19^, a large collection of SNP array and omics data for cancer cell lines. Also in this case by exploiting GWAS summary statistics trained in the TCGA dataset, CCLE germline genotype calls were used to calculate the 5 pPSS of interest across 995 CCLE cell lines. Somatic trait-specific aberrations for each cell line sample were determined considering (separately or in combination) reported somatic point mutations, homozygous deletions, and amplifications data identified within the corresponding oncogenic signaling pathways. For 2 of the 5 tested pPSS (40%) we found a statistically significant increase (*p*-value < 0.05) of pPSS distribution in CCLE samples harboring somatic trait-specific aberrations (Supplementary Data 10). We found, for example, that patients harboring homozygous deletions in the RTK RAS showed increased RTK RAS pPSS values (Fig. 6a, right) and that patients harboring point mutations in WNT signaling pathway showed increased WNT pPSS values (Fig. 6b, right).

### Validation of PSS in an independent cancer-specific dataset

**Fig. 7:**
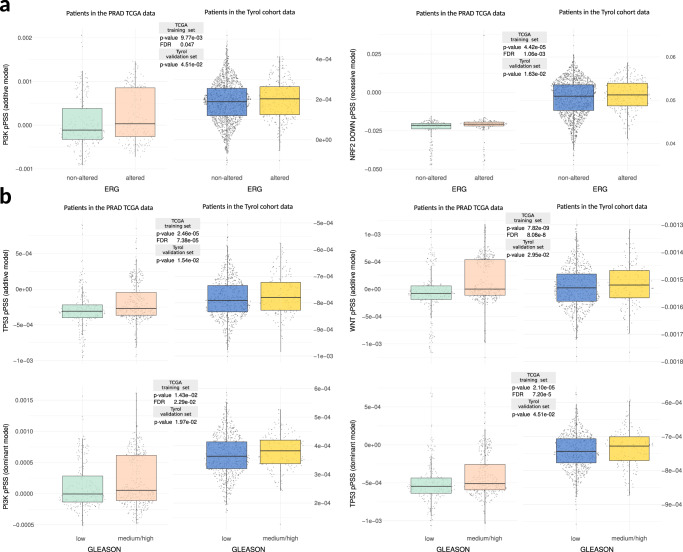
pPSS validation in a prostate cancer dataset. Boxplots showing statistically significant shift of pPSS distribution for ERG subtype (**a**) and in patients with moderate/high Gleason Score (GS) (**b**) in both TCGA dataset (left) and their confirmation in the Tyrol dataset (right). Kruskal–Wallis rank test sum was performed (two-tail test with FDR correction for TCGA and one-tail test for confirmation in the Tyrol dataset).

We finally evaluated our pPSS in the Tyrol cohort^28,29^, a prostate cancer (PCa) dataset including 1036 control samples and 837 cancer samples, of which 280 (of 492 with ERG gene status annotation) are annotated as PCa samples collected from patients overexpressing the ERG gene due to a TMPRSS2-ERG fusion (i.e., ERG subtype patients). Considering the effective ERG subtype classification performances that we observed in the TCGA PCa dataset (PRAD) for 5 pPSS, we tested to what extent this result could be validated in the Tyrol cohort. Exploiting GWAS summary statistics trained in the TCGA dataset, the 5 pPSS were calculated for all 837 cancer samples in the Tyrol dataset exploiting the available Tyrol genotype data. Two of the five pPSS (40%) also validated in the Tyrol cohort (Fig. 7a), and one demonstrated a similar (though not significant) trend. Notably, a logistic regression model built using the two validated pPPS demonstrated in the Tyrol cohort statistically significant performances (*p*-value = 0.033) in ERG subtype classification.

The Tyrol cohort provides also clinical information about patients’ Gleason Score (GS), a grading system representing one of the best independent predictor of prostate cancer clinical outcome^30^. Of the 19 pPSS that in the discovery TCGA dataset demonstrated a significant association with moderate/high-grade prostate cancer patients (i.e., patients with GS equal to 4 + 3 or greater than 7, respectively), four (21%) also validated in the Tyrol cohort (Fig. 7b) and one other demonstrated a similar (though not significant) trend.

Overall, the predictive power of pPSS was further validated in an independent cancer-specific dataset and we additionally demonstrated that pPSS could be effective in stratifying patients with more aggressive cancer phenotypes.

## Discussion

Over the past 15 years, despite numerous common SNPs have been linked by GWAS studies to the susceptibility of developing different cancer types, most of the identified associations demonstrated modest albeit significant effects. GWAS studies have been usually designed to measure the increased risk that individuals have in developing a specific cancer type. However, in the last ten years, cancer genomes studies based on next-generation sequencing data have unveiled how cancer is heterogeneous, characterized by the presence of multiple molecular subtypes and recurrently targeting signaling pathways and biological processes that are now recognized as hallmarks across many well-known forms of cancer.

This motived a deeper exploration of germline-somatic interactions, leading to a clear evidence that genetic background can influence the somatic evolution of tumors^10–14,31–34^. Here, we dug further into the exploration of this germline and somatic interplay, using a GWAS-based approach with additive and non-additive^35,36^ models and exploiting the availability of matched germline genotypes and somatic phenotypes from large-scale projects like TCGA, ICGC PCAWG, and CCLE. The datasets utilized in our analyses are multi-ancestry, with European ancestry being the dominant population. Although we employed logistic regression combined with principal component analysis instead of more advanced models, extensive evidence has demonstrated the effectiveness of our approach, particularly in the context of case-control studies^37–41^. Further, other recent GWAS studies successfully used logistic regression with PCA correction on TCGA data^31,42^.

Overall, we found evidence that germline genetics can influence the aberration of specific oncogenic signaling pathways, highlighting hence how individuals’ genetic background may contribute to the activity and stability of fundamental biological processes that are recurrently disrupted in cancer. A large fraction of the SNPs we found associated in our GWAS were indeed known *cis*-eQTLs of genes closely connected to oncogenes, tumor suppressor genes or cancer-related genes. In addition, we identified functional links between specific GWAS-associated SNPs and the corresponding oncogenic pathways traits, exploring for some of them putative biological interpretations that are in line with scientific knowledge and literature. As an example, we highlighted a SNP associated with NRF2 signaling pathway deregulation that is linked in *cis* to genes that are co-expressed with genes in the pathway across multiple tissues. Of note, the alternative allele of the SNP was indicative of a transcriptional signature associated with downregulation of KEAP1/CUL3/RBX1 complex, which acts as regulator of NRF2 levels in various cancers^43,44^.

The ability to analyze and integrate different matched omics data enabled us not only to identify and functionally characterize putative links between specific SNPs genotypes and the aberration of specific oncogenic signaling pathways, but also to exploit the theory of polygenic scores to investigate patients’ genetic liability to develop specific molecular profiles or particularly aggressive forms of cancer. While polygenic scores have been recently proven valuable in cancer risk prediction with multiple areas where they can have strong clinical utility, recent reports demonstrate that they can preferentially predict patients belonging to certain tumor subtypes or carrying specific somatic aberrations^45^, highlighting hence the importance to better understand their association with molecular and clinical variables. In line with this, our study demonstrates that individuals’ genetic background may influence the aberration of oncogenic processes in a way that is orthogonal with respect to the tumor type but important for specific tumor subtypes or to cancers that are particularly aggressive.

Our results are also in line with^10^, were the authors identified polymorphisms associated to specific tumor types or specific cancer driver gene alterations. While in both cases a genome-wide association approach was exploited to study germline-somatic links, our approach is substantially different. Indeed, we performed a pan-cancer analysis that explores germline-somatic links at the level of pathway, and in particular we investigated the polygenic nature of those links. Although, and as expected, we had no specific overlap with polymorphism reported in^10^, the two studies can be considered complementary, since by exploring different dimensions of germline-somatic links they both converge to the same conclusion that germline variants have a significant influence on specific somatic changes in tumors.

While the specific germline-somatic interactions we identified and reported may be used to generate testable hypothesis about mechanistic processes related to cancer genesis and progression, an important question would be to what extent our PSS could be useful in a clinical setting. Although the PPS we have studied demonstrated AUC below 0.8 (which represent a well-recognized threshold of high predictive power), some of our pan-cancer PSS were able to stratify patients based on OS and PFI in an extremely effective and cancer-specific manner. In addition, classification models built from our PSS demonstrated effective in identifying tumor subtypes and tumors with more aggressive phenotypes both in the discovery but also in external pan-cancer and cancer-specific datasets.

**Fig. 8:**
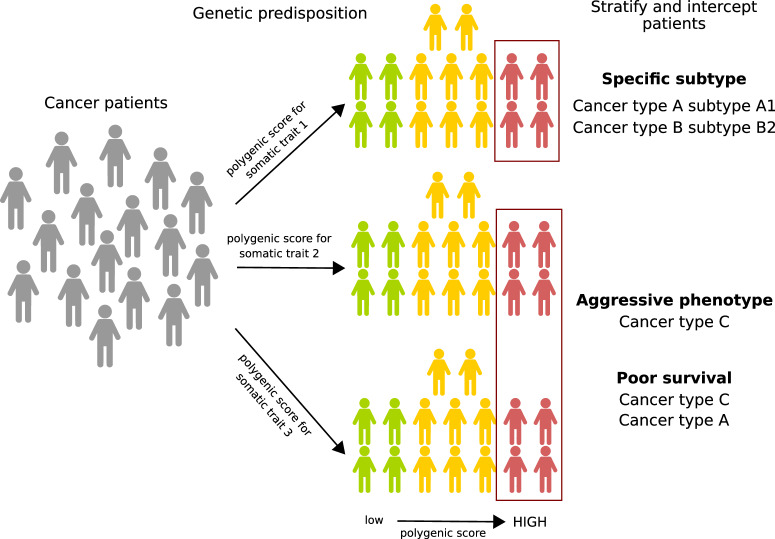
Polygenic scores model to describe patients’ genetic liability to develop specific cancer profiles. Cancer patients are stratified based on multiple polygenic scores built from somatic phenotypic traits. Somatic traits represent patients’ predisposition to carry somatic aberrations in specific oncogenic signaling pathways. Single polygenic scores or combination of polygenic scores can identify patients with more aggressive phenotypes, specific tumor subtypes or patients with poorer survival.

This study has several limitations, including the relatively small size of the TCGA dataset, the absence of an independent validation dataset with specular data characteristics, and the limited clinical utility that our OS and PFI results could have given that TCGA was not designed for clinical outcome studies. We, however, envision that our approach could be exploited and refined to intercept cancer patients with a genetic background that could more likely make their cancer evolve and progress towards specific molecular and clinical trajectories (Fig. 8).

We want to underline that due to the subtle links that can relate tumor types and pathway aberration profiles, no explicit inclusion of the tumor type in the association model was considered in the current study. Indeed, while it has been established that genetics influences tumor type formation^10^, the extent at which it can act as a collider or mediator variable with respect to pathway aberration profiles is not easily definable, and further investigations are required. Furthermore, an increased number of recessive associations, primarily involving downregulation traits with slightly elevated GIs, were observed. While an increased GI may suggest a polygenic trait^46^, the instability of OR estimations observed across these traits made characterizing most of them challenging in our polygenic analyses. This necessitates future efforts to delve deeper into their characterization and their role in cancer predisposition and evolution.

In addition, while in this study we focused on a set of phenotypic traits derived from the aberration profiles of specific signaling pathways, more advanced methods could be explored to define somatic traits, were cancer-specific disruption of specific biological processes could be identified by combining germline and somatic tumor omics data together with network data (e.g., gene networks, protein-protein interaction network)^47^.

Future large-scale studies collecting both germline and somatic omics data should continue to explore links between germline genetics and somatic variants with the ultimate goal of identifying cancer risk biomarkers.

## Methods

### Landscape of inherited SNPs in cancer patients

Genotype calls generated from Affymetrix SNP Array 6.0 intensities of normal (non-tumor) samples were retrieved from the TCGA legacy archive (portal.gdc.cancer.gov/legacy-archive). Each SNP was there annotated with an allele count (0 = AA, 1 = AB, 2 = BB, −1 = missing) and a confidence score between 0 and 1. Genotype calls with a score larger than 0.1 (corresponding to an error rate of >10%) were set to missing and the data was reformatted with PLINK v2^48^. Only autosomal SNPs were considered. Hardy-Weinberg equilibrium (HWE) was calculated across European individuals, selected based on the ancestry calls previously defined in^49^, and reported in Supplementary Data 11. Samples with SNP call rates <0.9 were discarded. Multi-allelic SNPs and SNPs with call rates <0.9, minor allele frequencies <0.01, or HWE test *p*-values < 1e-06 were discarded resulting in 842,108 SNPs across 10,755 TCGA samples. Considering that batch effects associated with groups of samples processed together (plate effects) can lead to a bias in the estimation of variants allele frequencies^50^, we then searched for the presence of variants displaying strong link with plate. In details, analysis of plates was performed stratifying samples by population (considering AFR, EUR, AMR, EAS, SAS major populations as annotated by EthSEQ^51,52^ in^49^, Supplementary Data 11) and, for each population, comparing all samples of a particular plate with all other plate’s samples pooled together. Each variant was tested for the enrichment of genotypes in specific plates (across 275 plates) performing Fisher exact test considering additive, dominant, and recessive models. We discarded all the SNPs demonstrating a strong plate association (*p*-value < 1e-08) in at least one population and one statistical model, retaining however variants associated with 4 or more plates. In addition, we searched for variants showing links with specific tumor types using a procedure that is similar to the one used for plate association analysis. All the variants displaying a strong association (*p*-value < 1e-08) in at least one population and one statistical model with exactly one tumor type were excluded. Overall, genotype calls of 833,130 SNPs across 10,755 TCGA samples were finally considered. Principal Component Analysis (PCA) was performed on the final data using the *smartpca* function implemented in the EIGENSOFT tool^53^ and the first 6 components were extracted.

### GWAS traits definition

A set of phenotypic binary traits were defined based on the somatic aberration profiles corresponding to 10 oncogenic signaling pathways characterized in^20^ using TCGA data. The considered oncogenic pathways include Cell Cycle, HIPPO, MYC, NOTCH, NRF2, PI3K, RTK RAS, TGF Beta, TP53 and WNT (Supplementary Data 12). A set of phenotypic binary traits (referred to as *somatic traits*) were defined based on the somatic aberration profiles described in^20^, one for each oncogenic pathway considered. Figure 1a shows an example, based on TP53 pathway, of how a somatic trait is built. An additional set of phenotypic binary traits (referred to as *somatic transcriptomic traits*) were defined based on the expression deregulation profile of the list of genes defined in^20^ for each oncogenic pathway (Supplementary Data 12). Specifically, mRNA expression z-scores (RNASeq V2 RSEM) were retrieved from The cBioPortal for Cancer Genomics^54,55^ for each patient and an oncogenic pathway was considered up-regulated, down-regulated, or generally deregulated if at least two genes in the pathway had, respectively, an expression z-score >2, <−2 or not in the range [−2,2]. Supplementary Fig. 1a provides an example of how a somatic transcriptomic trait is built, with the TP53 pathway serving as an example. Overall, we defined 10 *somatic traits* and 30 *somatic transcriptomic traits*.

### GWAS association analysis

GWAS analyses were performed for each considered trait within the TCGA dataset. Associations of SNPs and traits were performed with PLINK v2 using logistic regression with firth-fallback parameter active, indicating that firth regression is used when logistic regression fails. The analyses were performed using age at diagnosis, sex and the first 6 principal components previously calculated as covariates. Of note, the selection of the number of principal components (PCs) was based on the observation that the first six were sufficient to capture all TCGA populations and subpopulations described in^49^. PCs 1–3 captured the major population structure, while PCs 4–6 captured Asian and European substructures (Supplementary Fig. 3). In addition, considering that in our scenario the assumption that the likelihood of a patient to have an oncogenic pathway altered is proportional to the number of alternative alleles may not be sufficient to explain the complex genetic architecture of cancer, all three additive, dominant, and recessive models were investigated. Overall, 8860 patients with phenotype and covariate data available were used in the analyses. Associations were calculated against the minor allele. Family structure in the analysis was controlled excluding 178 samples representing potential 3^rd^ degree relatives using a scaled KING kinship coefficient of 0.0422 (--king-cutoff parameter was used while running the analyses). We extracted all associations that achieved a genome-wide statistical significance threshold of *p*-value < 4.2e-10 (Bonferroni correction, adjusted also for the number of traits and models tested, i.e., 5e-08/120), but also suggestive associations considering a weaker threshold of *p*-value < 1e-06. The latter threshold was chosen, similar to^31^, based on the observation that our analyses were conducted across correlated traits (Supplementary Fig. 8), involving hundreds of thousands of SNPs (many of which in linkage disequilibrium), and encompassing both additive and non-additive dependent models. Associations flagged by PLINK as UNFINISHED were excluded from reported results. Cross-cancer heterogeneity of the resulting associated variants was determined calculating the ${I}^{2}$ index. In detail, the set of significant associations were tested again in each tumor type separately. The analyses were performed with PLINK as described before. GWAS summary statistics were combined via meta-analysis across tumor types using PLINK. Associations flagged by PLINK as UNFINISHED were not considered in the meta-analyses. Heterogeneity values ${I}^{2}$ were extracted and collected.

### Functional characterization of associated variants

For each GWAS (both genome-wide and suggestive) associated SNP, we identified all SNPs in strong linkage disequilibrium (LD) with them within a genomic window of 250 kb centered around the SNP. LD data was retrieved from the ENSEMBL database. Strong LD was defined as *R*^2^ > 0.8 and D‘ = 1. This extended list of associated SNPs and LD SNPs was then queried for genomic overlaps with regulatory elements, cancer genes, oncogenes, or tumor suppressor genes, and their disruptive effect on transcription factor binding motifs. Oncogenes (OGs, *N* = 82), tumor suppressor genes (TSGs, *N* = 63) and more generally cancer-related genes (*N* = 920) were characterized using a comprehensive list we compiled from literature. Regulatory elements for promoters, enhancers and active enhancers were retrieved using our resource CONREL^23^, while the impact of SNPs on putative transcription factor DNA binding motifs was retrieved from our resource Polympact^24^, which characterizes the impact of >18 million common SNPs across >5000 DNA motifs. SNPs were classified as disruptive when causing an absolute relative change of motifs’ score >0.5.

### Integrated protein-protein interaction network

A reference protein-protein interaction (PPI) network was built by merging information of five databases: BioGRID release 3.5.173^56^; HPRD release 9 20100413^57^; IntAct release 20150120^58^; BioPlex 3 release 20190502^59^; STRING release v11.0^60^. Interactions between nodes that represent human proteins and experimentally validated were retained. Predicted data, such as evolutionary analysis, gene expression data, and metabolic associations, were excluded. Interactions from STRING and IntAct databases were filtered considering only interactions with reported confidence scores higher than 700 and 0.6 respectively. Interactions from BioGRID, HPRD, and BioPlex were all included because manually curated. After the removal of duplicated edges, the resulting network contains 245,787 interactions and 16,514 unique human proteins.

### *Cis*-eQTL and co-expression analyses

GTEx v8 RNAseq count matrices were downloaded from recount3 database^61^. For each tissue, logarithm (two-based) transformed RPKM + 1 of each gene was calculated using R *recount* and *recount3* packages and quantile normalized using R *limma* package^62^. A total of 16,805 RNA-seq samples across 42 tissues were used in the analysis. *cis*-eQTL data for GWAS SNPs (both genome-wide and suggestive) were retrieved from GTEx data portal (gtexportal.org). SNP/gene *cis*-eQTL links were stratified by tissue and for each tissue *cis*-eQTL genes in that tissue were collected and tested for co-expression against all other protein-coding genes expressed in the same tissue, using Pearson correlation and correcting *p*-values with FDR method. Only correlation values smaller than −0.50 or greater than 0.50 and with FDR < 0.05 were considered significant.

### Polygenic somatic scores construction

For each considered trait, a set of polygenic scores were computed using a five-fold cross-validation approach and exploiting the TCGA dataset. TCGA samples were randomly partitioned into five equal-sized disjoint subsets. For each fold, a partition was retained as validation set while the others were aggregated and used as training set. A set of GWAS runs was performed in the training sets as previously described. Specifically, logistic regression was used, considering additive, dominant, and recessive models, and using age at diagnosis, sex, and the first 6 principal components as covariates. The generated GWAS summary statistics were then used in the validation set to build polygenic scores, referred to as polygenic somatic scores (PSS). PSS were calculated as the average number of minor alleles weighted by the allele’s effect size using PRSice-2^63^. As shown in^64,65^, using a more liberal but optimized *p*-value threshold instead of a genome-wide significant threshold, improves performance of polygenic scores prediction. Hence, a computational workflow was designed to build effective traits’ PSS and test their performances and statistical significance. As described in Supplementary Fig. 9, for each trait we first used PRSice-2 to determine the best *p*-value threshold (testing *p*-values ranging from 1e-08 to 1 and using a 1e-08 step) across different LD clumps (using *R*^2^ of 0.2, 0.4, 0.6, 0.8 and 1). In particular, to determine the optimal *p*-value threshold for each clump, we averaged the p-value thresholds at the highest pseudo-*R*^2^, when significant (*p*-value < 0.05), that we obtained across the five folds. Then, we used PRSice-2 again to generate for each LD clump a trait’s score using the corresponding best *p*-value threshold and calculating its representative AUC performance score, which was obtained averaging the AUC values obtained across the five folds (R *pROC* package^66^ was used to compute the AUCs). This to finally select the best-performing combination of *p*-value threshold and LD clump that was used to generate the trait’s PSS. Further, to better characterize the statistical significance of PSS performances, we implemented an additional analysis step that is based on permutation analysis. In detail, for each of the 120 PSS (40 traits across 3 association models), 100 random PSS were generated by randomly shuffling trait’s labels, and for each of them performances in terms of AUC values were computed using the same computational workflow described before, producing a PSS’s specific AUC baseline reference distribution. Then, for each PSS the observed AUC value and the corresponding AUC baseline reference distribution were used to compute an empirical *p*-value. Specifically, each empirical *p*-value was computed as (*r* + 1)/(*n* + 1), where n is the size of the reference distribution and *r* is the number of AUC values in the reference distribution that are greater or equal to the observed AUC. *P*-values were finally corrected for multiple hypothesis testing using FDR method. A set of *pan-cancer PSS (pPSS)* was finally defined only considering PSS with an FDR < 0.25.

### Survival analysis

TCGA survival data was retrieved from^27^. Overall survival (OS) and Progression-Free Interval (PFI) data were used. Survival analysis was performed to examine to what extent clinical endpoints correlate with both the somatic (transcriptomic) traits and pPSS within individual tumor types. Also in this case, a five-fold cross-validation approach was applied. Analysis was performed using the R *survival* package^67^. For the analysis based on somatic (transcriptomic) traits, patients were stratified based on traits definitions. For pPSS analysis, patients were grouped and tested on the median value of each selected pPSS. In detail, for each fold analysis, a Cox proportional hazards regression model was computed in the training set and then used in the validation set to compute the performance (AUC) which evaluates the ability of the model to discriminate patients with altered pathways or the patients with a higher pPSS. Also in this case, the performances of our survival models were compared against AUC baseline reference distributions generated by permutation analyses. Empirical *p*-values were computed as described previously. For both analyses, OS and PFI associations were corrected for multiple hypotheses separately and for each tumor type. OS and PFI associations with an FDR < 0.25 for both somatic (transcriptomic) traits and pPSS analyses were highlighted.

### Analysis of tumor subtypes

TCGA cancer subtypes were collected from^49^. A total of 5148 samples were annotated with molecular subtypes for the following tumor types: BLCA, BRCA, CESC, COAD, ESCA, GBM, HNSC, LGG, READ, SARC, STAD, TGCT, and UCEC. The molecular subtypes of TCGA prostate cancer (PRAD) dataset were retrieved from^12^. Only TCGA patients included in our polygenic scores computations were retained and then tumor subtypes with less than 20 patients were discarded. A total of 4818 patients, representing 13 tumor types spanning more than 40 different tumor subtypes, were used in the analysis. For each tumor type, we tested the presence of significant deviation in the distribution of pPSS across different tumor subtypes applying a five-fold cross-validation approach as described previously. In detail, for each combination of tumor subtype and pPSS, statistical significance was determined building a logistic regression model in the training set testing all samples of a particular tumor subtype against all other tumor samples of that tumor type. Then, the performance (AUC) of the model was computed in the validation set. Also in this case, the performances of our models were compared against AUC baseline reference distributions generated by permutation analyses. An empirical *p*-value for each combination of pPSS and tumor subtype was calculated as described previously. For each tumor subtype, associations were corrected for multiple hypotheses. Given the non-standard u-shape distribution of *p*-values that we observed for some combinations, associations were here corrected using the robust FDR method described in^68^. Only FDR < 0.25 were considered significant. For each tumor subtype, significant pPSS were integrated using a logistic regression model to test their predictive power in identifying tumor subtypes.

### Validation using PCAWG data

Data for somatic point mutations, somatic copy number aberrations, together with matched common SNPs genotype calls and relevant clinical information were obtained from the ICGC PCAWG project^17^ for 1823 patients. Based on available samples annotations, samples that are both in TCGA and ICGC projects were not considered in the analysis. Genotyping files (VCF format) representing a total of 67,207,291 germline variants were downloaded from the ICGC Data Portal (dcc.icgc.org). INDELS and SNPs not in the TCGA genotype dataset were excluded. A total of 830,168 variants were retrieved and used to build pPSS exploiting the weights previously trained in the TCGA dataset. Specifically, scores were calculated with PRSice-2 using TCGA GWAS summary statistics filtered based on PSS TCGA-specific optimal *p*-value thresholds and LD clump cutoffs. Somatic point mutations and somatic copy number aberrations were downloaded for each patient and used to collect somatic trait-specific genomic aberrations. Specifically, for each gene in a somatic trait defined by an oncogenic signaling pathway, we retrieved non-synonymous point mutations, homozygous deletions, and amplifications. We considered only the somatic copy number aberrations consistent with the role of the gene (deep deletion of TSGs and amplification of OGs, as defined above). Somatic alterations data representing the presence of gene aberration were integrated and summarized across patients. Due to the differences between data in TCGA and ICGC PCAWG projects, aberrations were not aggregated but kept separated. Binary somatic trait-specific aberration profiles were defined for each patient considering separately or in different combinations the three types of somatic aberrations. Distributions of pPSS in the different groups were compared using Wilcoxon-test statistics (two-tail) and *p*-values were corrected for multiple hypotheses. Only results with FDR < 0.25 were considered significant.

### Validation using CCLE data

Data for somatic point mutations, somatic copy number aberrations, together with matched SNP Affymetrix 6.0 array Birdseed calls were obtained from the CCLE data portal for 995 cell lines^18,19^. Each SNP was there annotated with an allele count (0 = AA, 1 = AB, 2 = BB, −1 = missing) and a confidence score between 0 and 1. Genotype calls with a score larger than 0.1 were set to missing and the data were reformatted with PLINK v2^48^. A total of 868,261 variants were retrieved and used to build pPSS exploiting the weights previously trained in the TCGA dataset. As for ICGC, scores were calculated with PRSice-2 using TCGA GWAS summary statistics filtered based on PSS TCGA-specific optimal *p*-value thresholds and LD clump cutoffs. Somatic point mutations and somatic copy number aberrations were downloaded for each cell line and used to collect somatic trait-specific genomic aberrations. Data was processed as described in the previous section. Only pPSS resulting significant in the ICGC validation were tested for confirmation in CCLE data using a Wilcoxon-test statistic (one-tail) with 0.05 *p*-value cutoff.

### Validation using Tyrol cohort data

SNP genotype calls (Affymetrix SNP Array 6.0) data and clinical information for 1903 individuals from the Tyrol Early Prostate Cancer Detection Program cohort were retrieved from^28,29^. The data include genotype calls for 1036 healthy control individuals and 867 prostate cancer (PCa) patients. Of these, 492 had annotation for ERG status with 280 patients (57%) annotated as positive for the TMPRSS2-ERG fusion (ERG subtype patients). In addition, 159 patients were annotated as having a moderate/high Gleason Score (GS) of 4 + 3 (N = 54) or >7 (*N* = 105). A total of 871,856 SNPs were retrieved and used to build pPSS exploiting the weights previously trained in the TCGA dataset. Also in this case, scores were calculated with PRSice-2 using TCGA GWAS summary statistics filtered based on PSS TCGA-specific optimal *p*-value thresholds and LD clump cutoffs. Only pPSS resulting significant (FDR < 0.25) in the TCGA PRAD subset were tested for confirmation in the Tyrol dataset. Distributions of PSS were compared using Wilcoxon-test statistic (one-tail) to identify PCa ERG subtype patients and patients with high GS with 0.05 *p*-value cutoff. Significant pPSS were integrated using a logistic regression model to test their predictive power in identifying ERG-positive patients.

### Reporting summary

Further information on research design is available in the Nature Research Reporting Summary linked to this article.

### Supplementary information

REPORTING SUMMARY

Supplementary Figures

Supplementary Data

## Data Availability

The data generated in this study are available within the article and its supplementary data files. Tyrol cohort data access and use were granted by the lead contacts of the corresponding studies. All other human and cell line data used in this study come from publicly available sources, however some of these sources require controlled access. The raw data can be obtained directly from the source studies. The processed form of the data used to support the findings of this study are available on request from the corresponding author AR. Because many of the sources are controlled access, the requestor must have approved access for the data to be shared. For Data Access to processed genotyping and transcriptomic data, contact corresponding author with proof of access to dbGaP studies where controlled access is required. More information for controlled access dataset is available for TCGA (https://docs.gdc.cancer.gov/Data/Data_Security/Data_Security/) and ICGC PCAWG (https://docs.icgc.org/pcawg/data/).

## Code availability

This study didn’t generate original codes. Any additional information required to reanalyze the data reported in this paper is available from the lead contact upon request.
