# Supplementary material for: Germline determinants of aberrant signaling pathways in cancer
Source: NPJ Precis Oncol. 2024 Mar 1;8:57. doi: 10.1038/s41698-024-00546-5 (PMC10907629; doi:10.1038/s41698-024-00546-5)
Supplement: Supplementary file 1 — REPORTING SUMMARY [file 41698_2024_546_MOESM1_ESM.pdf]

nature portfolio

Corresponding author(s): Alessandro Romanel

Last updated by author(s): Oct 27, 2023

Reporting Summary

Nature Portfolio wishes to improve the reproducibility of the work that we publish. This form provides structure for consistency and transparency in reporting. For further information on Nature Portfolio policies, see our Editorial Policies and the Editorial Policy Checklist.

Statistics

For all statistical analyses, confirm that the following items are present in the figure legend, table legend, main text, or Methods section.

n/a	Confirmed	
☐	☒	The exact sample size (*n*) for each experimental group/condition, given as a discrete number and unit of measurement
☐	☒	A statement on whether measurements were taken from distinct samples or whether the same sample was measured repeatedly
☐	☒	The statistical test(s) used AND whether they are one- or two-sided<br>*Only common tests should be described solely by name; describe more complex techniques in the Methods section.*
☐	☒	A description of all covariates tested
☐	☒	A description of any assumptions or corrections, such as tests of normality and adjustment for multiple comparisons
☐	☒	A full description of the statistical parameters including central tendency (e.g. means) or other basic estimates (e.g. regression coefficient) AND variation (e.g. standard deviation) or associated estimates of uncertainty (e.g. confidence intervals)
☐	☒	For null hypothesis testing, the test statistic (e.g. *F*, *t*, *r*) with confidence intervals, effect sizes, degrees of freedom and *P* value noted<br>*Give P values as exact values whenever suitable.*
☒	☐	For Bayesian analysis, information on the choice of priors and Markov chain Monte Carlo settings
☒	☐	For hierarchical and complex designs, identification of the appropriate level for tests and full reporting of outcomes
☐	☒	Estimates of effect sizes (e.g. Cohen's *d*, Pearson's *r*), indicating how they were calculated

Our web collection on statistics for biologists contains articles on many of the points above.

Software and code

Policy information about availability of computer code

Data collection	For TCGA, genotype calls data was retrieved from the TCGA legacy archive (portal.gdc.cancer.gov/legacy-archive). Somatic mutations and mRNA expression z-score were retrive from cBioPortal using cgdsr (v.1.3.0) R package. GTEx v8 RNAseq count matrices were downloaded using recount3 (v1.6.0) R package.<br>Genotype calls and somatic aberrations data for CCLE and ICGC PCAWG cohorts were downloaded from the CCLE Data Portal (depmap.org/portal/) and the ICGC Data Portal (dcc.icgc.org), respectively.
Data analysis	All software and code used in this study have been described in published literature. R (v3.6.3 and v4.2.2), PLINK (v2.00a3LM, www.cog-genomics.org/plink/2.0/). PRSice-2 (v2.3.3, choishingwan.github.io/PRSice/), EIGENSOFT tool (v5.0.2, github.com/DReichLab/EIG), EthSEQ (v2) R package, recount3 (v1.6.0) R package, limma (v3.52.4) R package, pROC (v1.18.4) R package, survival (v3.4-0) R package, stats (v4.2.2) R package, prot2D (v1.20.0) R package, igraph (v1.5.1) R package.

For manuscripts utilizing custom algorithms or software that are central to the research but not yet described in published literature, software must be made available to editors and reviewers. We strongly encourage code deposition in a community repository (e.g. GitHub). See the Nature Portfolio guidelines for submitting code & software for further information.

Data

Policy information about availability of data

All manuscripts must include a data availability statement. This statement should provide the following information, where applicable:

- Accession codes, unique identifiers, or web links for publicly available datasets
- A description of any restrictions on data availability
- For clinical datasets or third party data, please ensure that the statement adheres to our policy

Additional information on accessing the data, including raw read files, can be found at https://docs.gdc.cancer.gov/Data/Data_Security/Data_Security/ and https://docs.icgc.org/pcawg/data/. In accordance with the data access policies of the ICGC and TCGA projects, most molecular, clinical and specimen data are in an open tier which does not require access approval. To access potentially identification information, such as germline alleles and underlying sequencing data, researchers will need to apply to the TCGA Data Access Committee (DAC) via dbGaP (https://dbgap.ncbi.nlm.nih.gov/aa/wga.cgi?page=login) for access to the TCGA portion of the dataset (dbgap accession: phs000178.v11.p8), and to the ICGC Data Access Compliance Office (DACO; http://icgc.org/daco) for the ICGC portion. GTEx v8 RNAseq data were downloaded from GTEx Portal (gtexportal.org). Tyrol Early Prostate Cancer Detection Program cohort data were retrieve from [PMID: 22496589].

Research involving human participants, their data, or biological material

Policy information about studies with human participants or human data. See also policy information about sex, gender (identity/presentation), and sexual orientation and race, ethnicity and racism.

Reporting on sex and gender	Both female and male sex partecipants were included in analysis, and sex was included as covariates in training and validation analyses. Findings are applicable to both sex.
Reporting on race, ethnicity, or other socially relevant groupings	Individuals from the 5 major super-populations (AFR, AMR, EAS, EUR, SAS) were included and used for training and validation analyses. In all studies, European was the dominant population. The first 6 principal components were used as covariates in all analyses.
Population characteristics	*Describe the covariate-relevant population characteristics of the human research participants (e.g. age, genotypic information, past and current diagnosis and treatment categories). If you filled out the behavioural & social sciences study design questions and have nothing to add here, write "See above."*
Recruitment	*Describe how participants were recruited. Outline any potential self-selection bias or other biases that may be present and how these are likely to impact results.*
Ethics oversight	*Identify the organization(s) that approved the study protocol.*

Note that full information on the approval of the study protocol must also be provided in the manuscript.

Field-specific reporting

Please select the one below that is the best fit for your research. If you are not sure, read the appropriate sections before making your selection.

☒ Life sciences ☐ Behavioural & social sciences ☐ Ecological, evolutionary & environmental sciences

For a reference copy of the document with all sections, see nature.com/documents/nr-reporting-summary-flat.pdf

Life sciences study design

All studies must disclose on these points even when the disclosure is negative.

Sample size	This study required matched genotype and phenotype information from available datasets, which was the limiting factor on sample size. TCGA was selected for training analysis as it is the largest dataset that provides genotype and phenotype information with tumor samples profiled for 10,755 samples. ICGC PCAWG was used as validation dataset
Data exclusions	Data exclusion criteria were defined in advance. For training, individuals who were related to each other (as determined by a KING kinship coefficient greater than 0.0422, which corresponds to third-degree relatedness) were excluded. Quality control criteria excluded individuals with genotype coverage less than 90%. For the ICGC PCAWG dataset, all individuals who were already included in the TCGA analysis were excluded.
Replication	Polygenic score and survival were all validated. Polygenic somatic scores were constructed using TCGA cohort and validated in ICGC PCAWG, CCLE and Tyrol cohorts. Survival was tested on mutational pathway status and validated on pPSSs stratification.
Randomization	Best practices for randomizing samples were followed. Training process is repeated 5 times in a randomized 5 fold cross-validation setup. Permutation analysis was conducted and described in more details in methods.
Blinding	Blinding was not applicable as data collection was not performed in study. Other than exclusion criteria described above, all data was analyzed and presented.

Reporting for specific materials, systems and methods

We require information from authors about some types of materials, experimental systems and methods used in many studies. Here, indicate whether each material, system or method listed is relevant to your study. If you are not sure if a list item applies to your research, read the appropriate section before selecting a response.

Materials & experimental systems

n/a	Involved in the study
☒	☐ Antibodies
☒	☐ Eukaryotic cell lines
☒	☐ Palaeontology and archaeology
☒	☐ Animals and other organisms
☒	☐ Clinical data
☒	☐ Dual use research of concern
☒	☐ Plants

Methods

n/a	Involved in the study
☒	☐ ChIP-seq
☒	☐ Flow cytometry
☒	☐ MRI-based neuroimaging

Plants

Seed stocks

Report on the source of all seed stocks or other plant material used. If applicable, state the seed stock centre and catalogue number. If plant specimens were collected from the field, describe the collection location, date and sampling procedures.

Novel plant genotypes

Describe the methods by which all novel plant genotypes were produced. This includes those generated by transgenic approaches, gene editing, chemical/radiation-based mutagenesis and hybridization. For transgenic lines, describe the transformation method, the number of independent lines analyzed and the generation upon which experiments were performed. For gene-edited lines, describe the editor used, the endogenous sequence targeted for editing, the targeting guide RNA sequence (if applicable) and how the editor was applied.

Authentication

Describe any authentication procedures for each seed stock used or novel genotype generated. Describe any experiments used to assess the effect of a mutation and, where applicable, how potential secondary effects (e.g. second site T-DNA insertions, mosiacism, off-target gene editing) were examined.
