# Supplementary material for: Germline determinants of aberrant signaling pathways in cancer
Source: NPJ Precis Oncol. 2024 Mar 1;8:57. doi: 10.1038/s41698-024-00546-5 (PMC10907629; doi:10.1038/s41698-024-00546-5)
Supplement: Supplementary file 2 — Supplementary Figures [file 41698_2024_546_MOESM2_ESM.pdf]

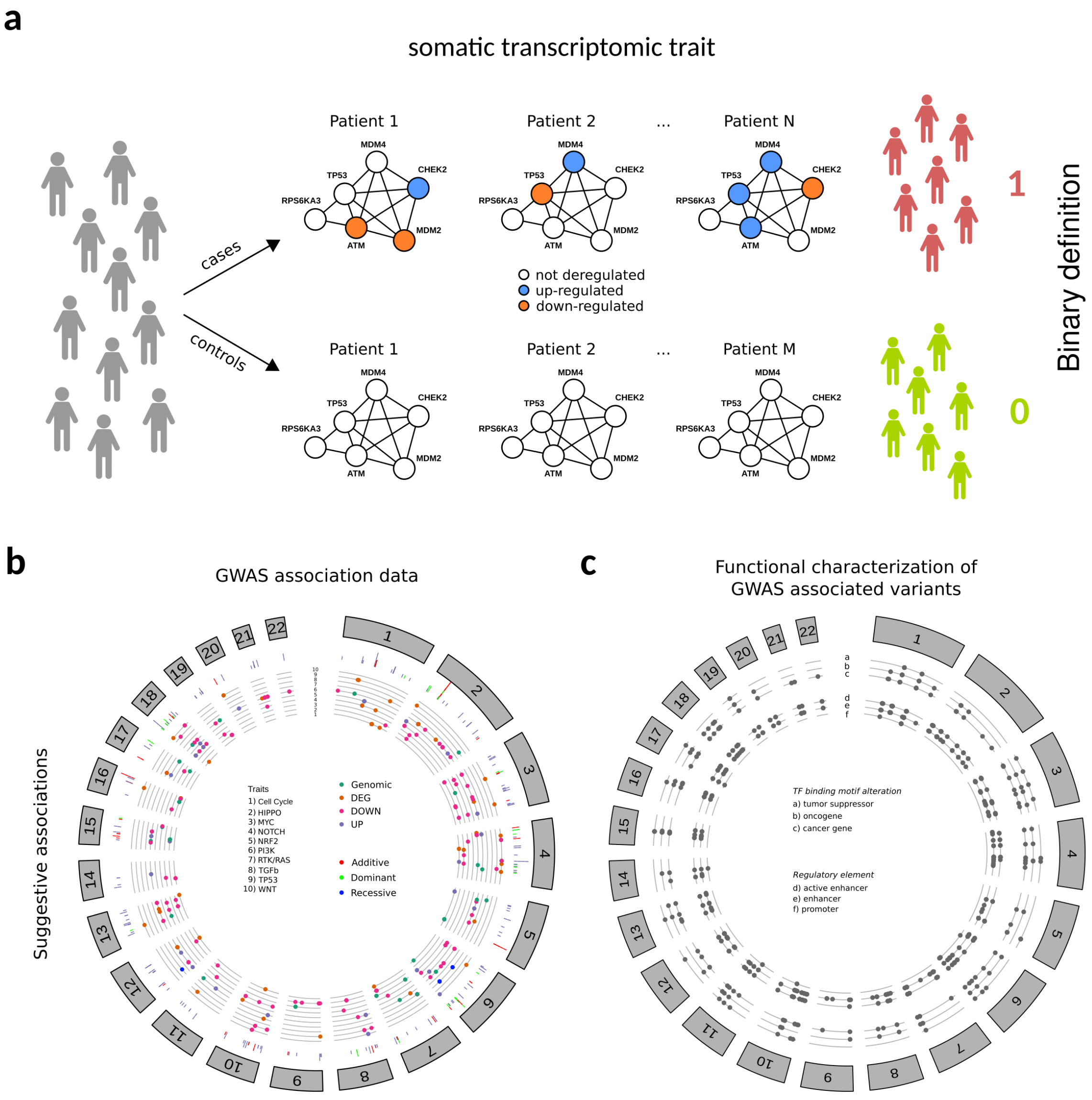

Supplementary Figure 1: Traits construction and GWAS results. a) Cancer patients are stratified based on the presence of transcriptomic alterations of genes in specific oncogenic signaling pathways to construct somatic transcriptomic binary traits. TP53 somatic transcriptomic trait is shown as an example of how the genes deregulation are used to build the trait. b) Circular plots showing GWAS results for suggestive significant associations with p-value in the range [1e-07,1e-06). The chromosomal positions (outer track) of the associations are shown for the forty traits in the inner track. The associations for different oncogenic pathways are reported on different rows and shown with different colors based on the trait's definition. In the middle track, the statistical models used for each association are shown in different colors. c) Circular plots showing functional characterization of suggestive associations with p-value in the range [1e-07,1e-06). The functional characterization is performed on LD extended associated variants. LD extended sets of associated variants are characterized for genomic overlaps with regulatory elements (inner track) and to cause a change in the transcription factor binding motifs of genes implicated in cancer (middle track). The chromosomal positions (outer track) are reported for the corresponding variant from the GWAS analyses.

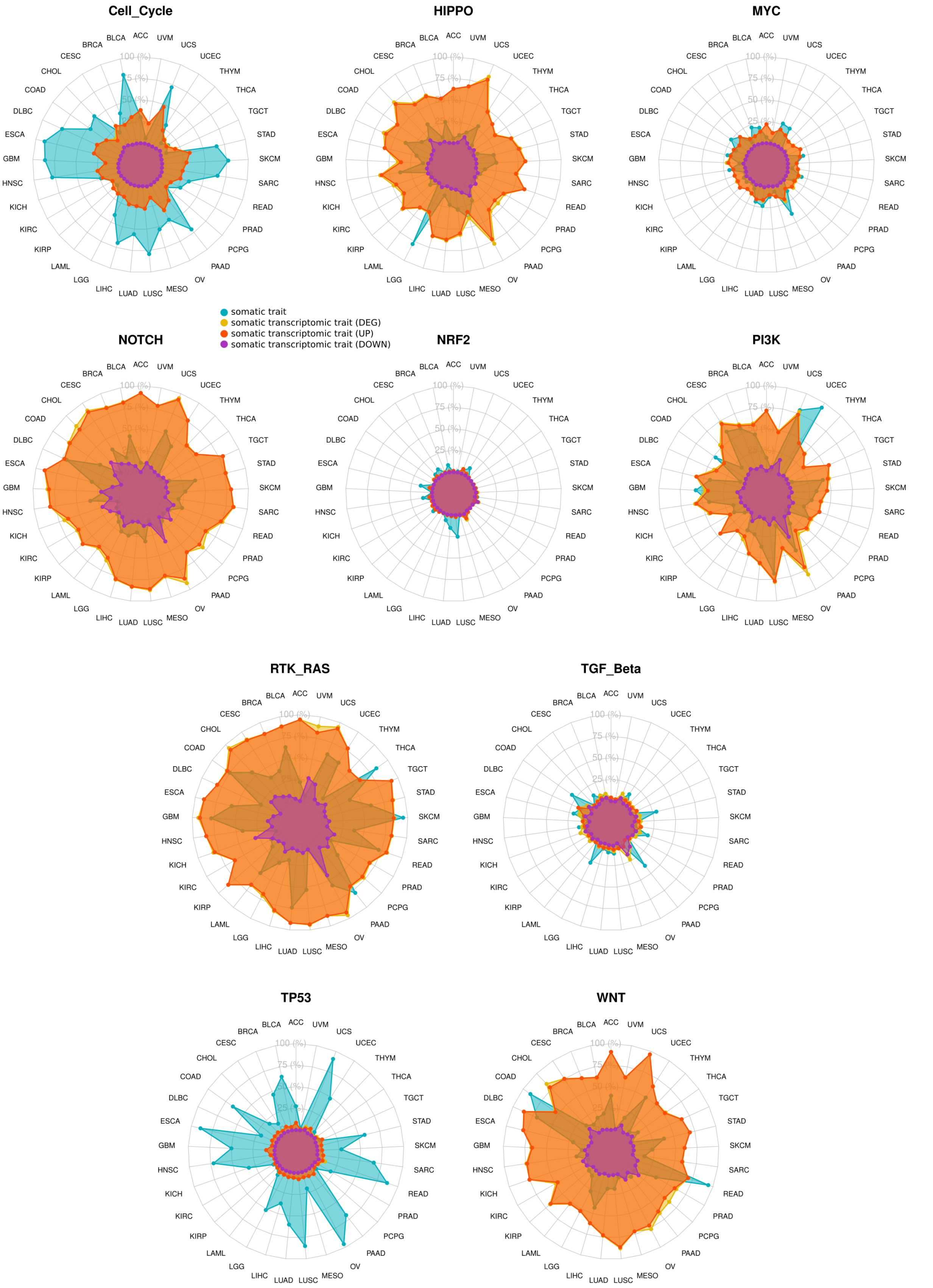

Supplementary Figure 2: Traits alteration frequencies. Radar plots showing the fraction of altered samples per trait across all tumor types.

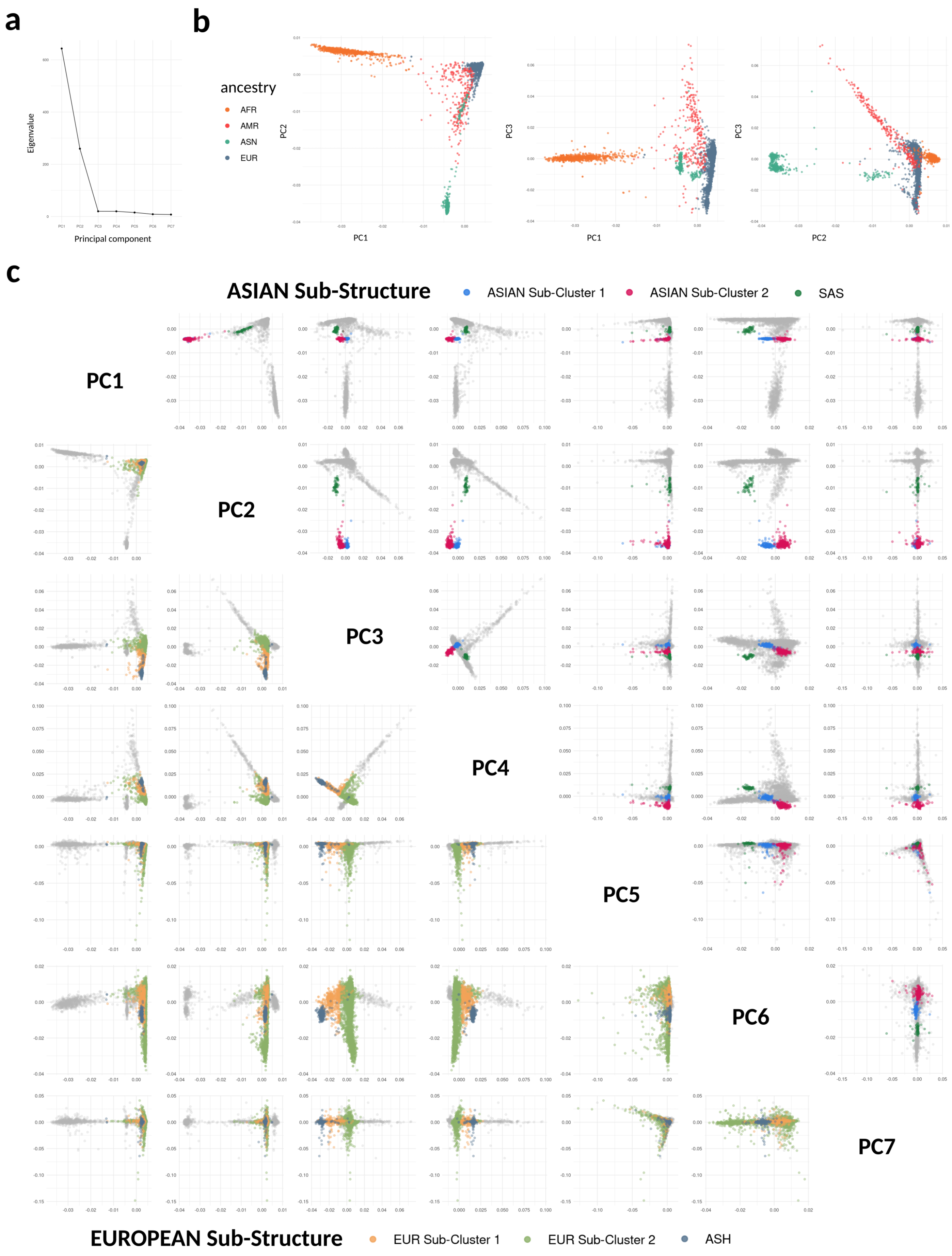

Supplementary Figure 3: Principal Component Analysis (PCA) and TCGA population structure. a) Scree plot of the first seven principal components (PCs); b) Major populations are captured by the first three PCs; c) Asian and European sub-populations are captured by the first six PCs. Annotations of populations and subpopulations are derived from (Carrot-Zhang et al., 2020).

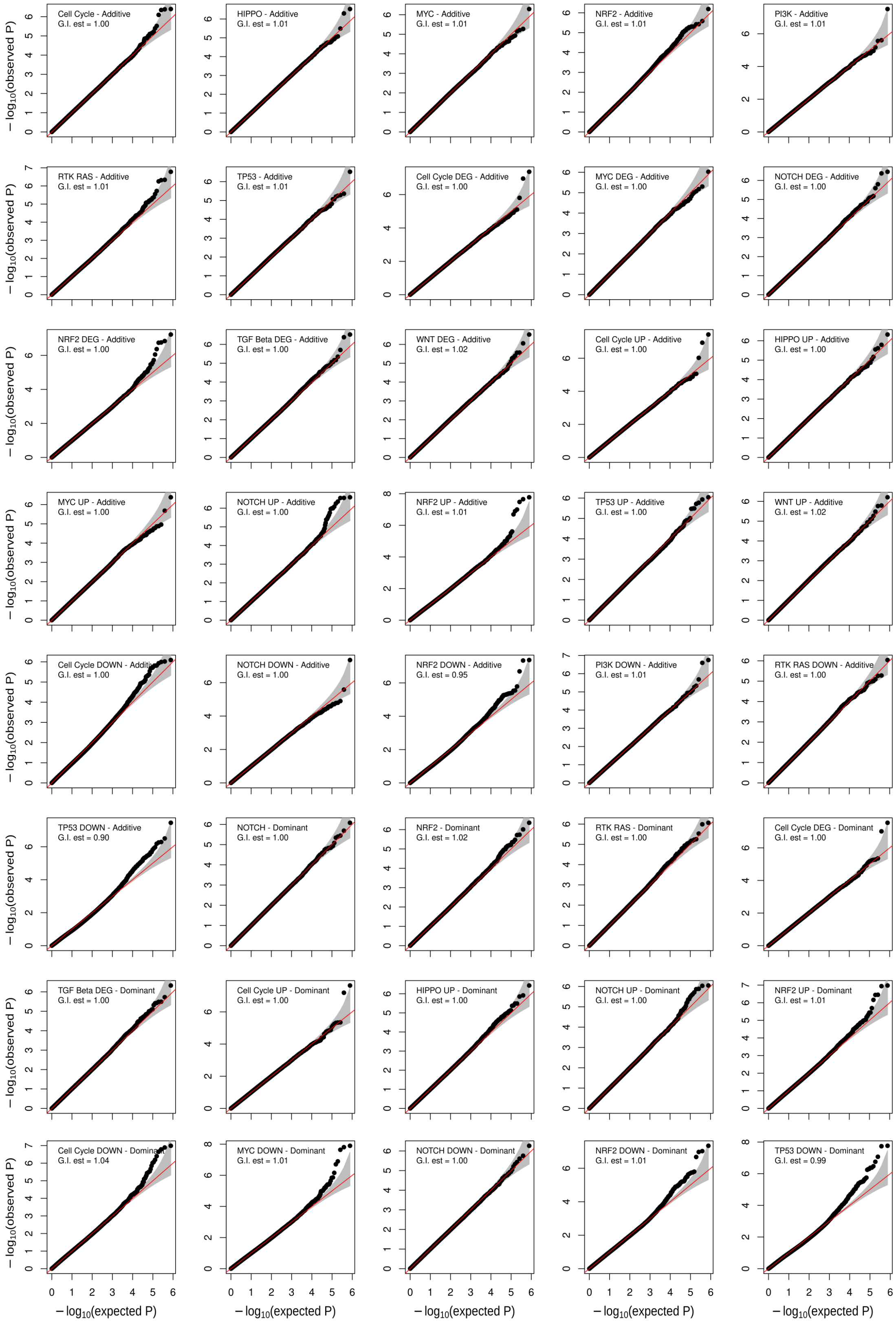

Supplementary Figure 4: Quantile-quantile (QQ) plots and genomic inflation (G.I.) estimates for GWAS with traits showing significantly associated SNPs. Red lines represent the expected distributions, the 95% confidence interval is shaded in gray.

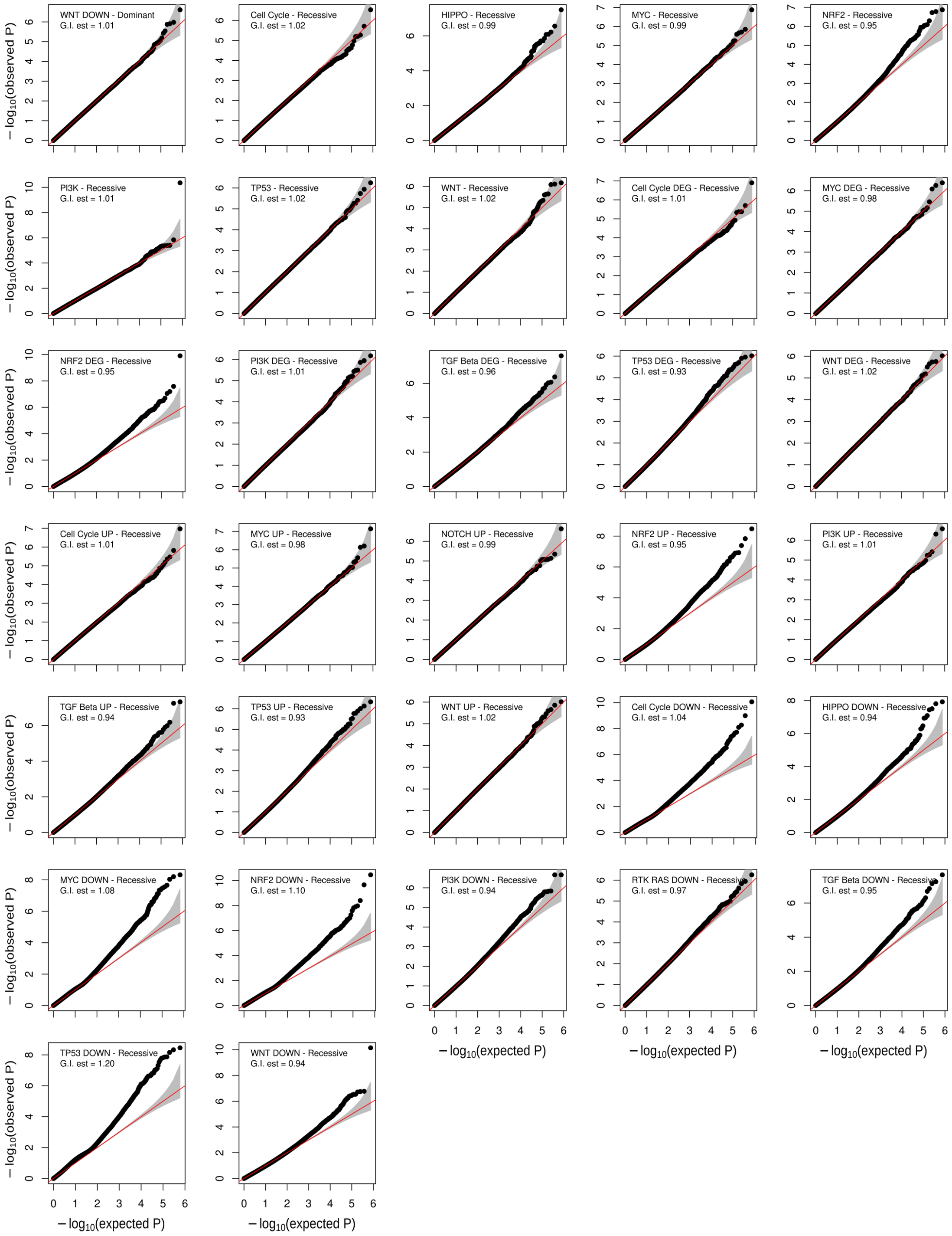

Supplementary Figure 4: (see legend in previous page)

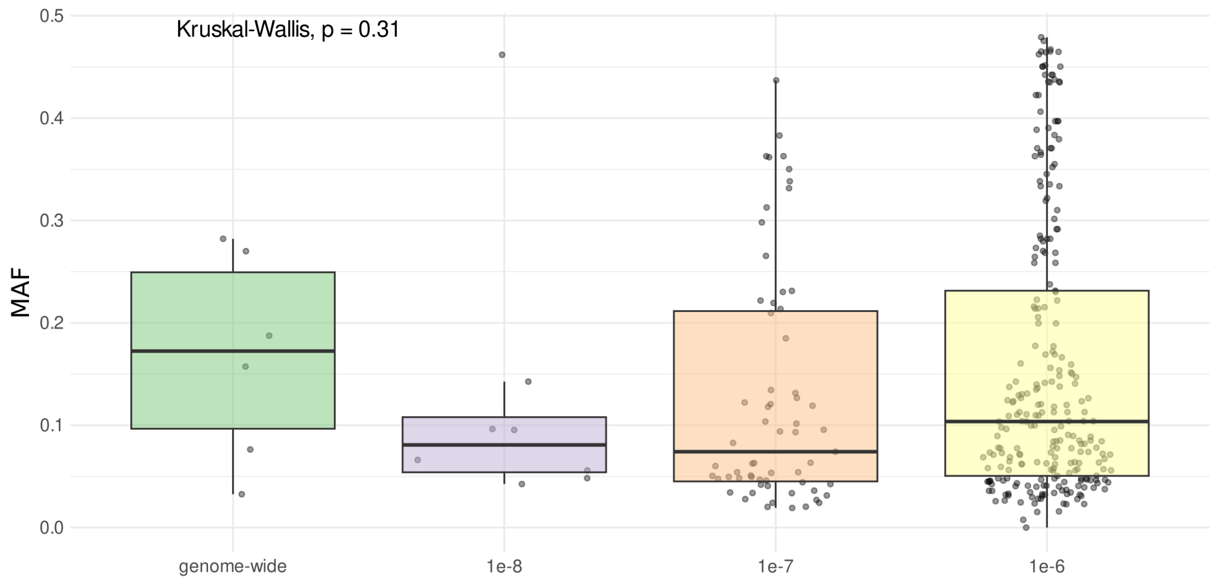

Supplementary Figure 5: Boxplots showing the distributions of the Minor Allele Frequencies (MAFs) of genome-wide and suggestive (<1e-8, <1e-7, <1e-6 p-value thresholds) associated SNPs.

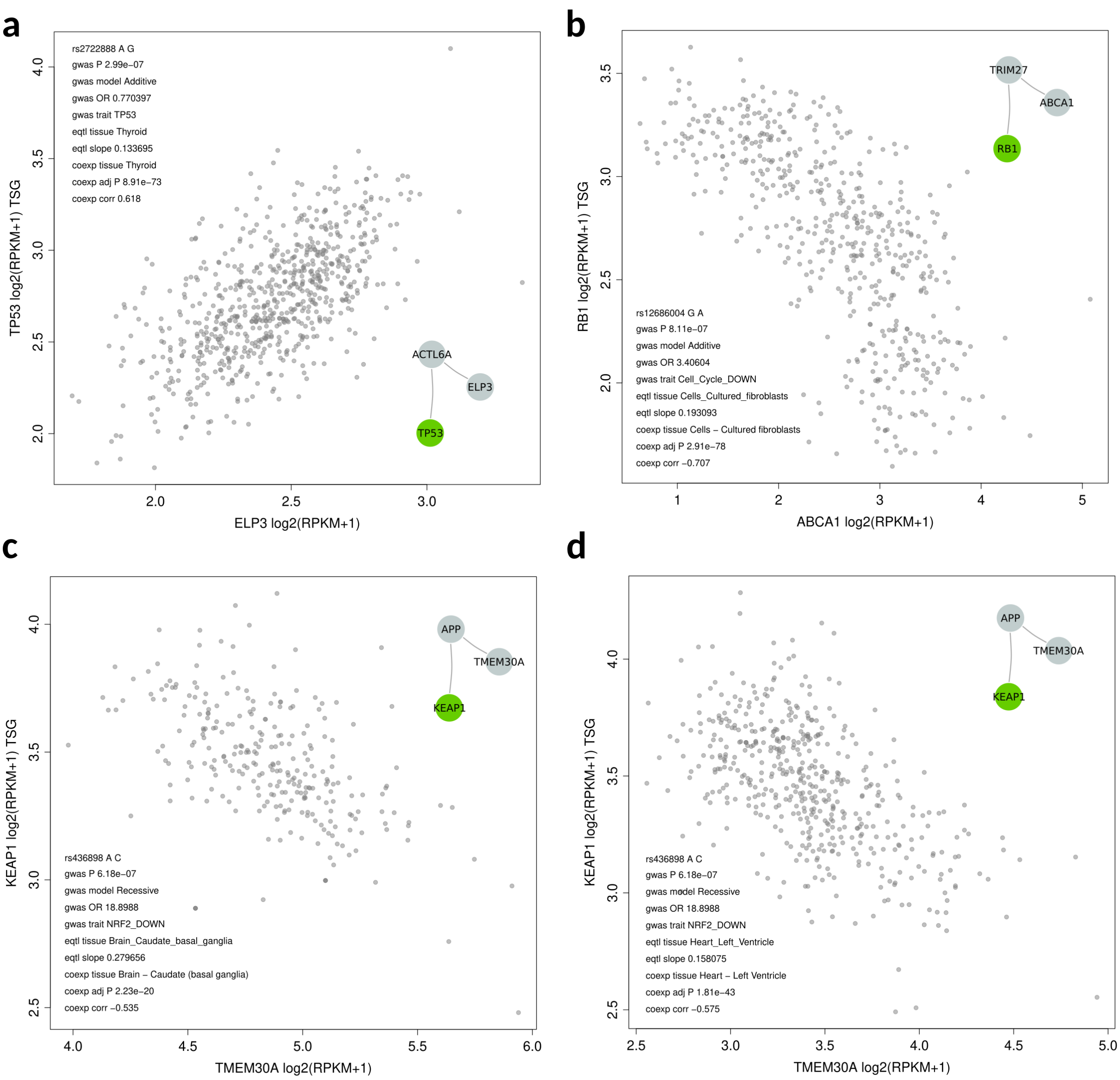

Supplementary Figure 6: Examples of cis interactions with genes that are co-expressed with genes in the oncogenic pathways of the corresponding traits. These putative links are supported by a close link (PPI distance 2) between the two proteins. a) shows the co-expression between cis-eQTL ELP3 gene of the variant rs2722888 (found associated with TP53 somatic trait) and TP53 gene. b) shows the co-expression between cis-eQTL ABCA1 gene of the variant rs12686004 (found associated with Cell cycle somatic transcriptomic DOWN trait) and RB1 gene. c-d) shows the co-expression between cis-eQTL TMEM30A gene of the variant rs436898 (found associated with NRF2 somatic transcriptomic DOWN trait) and KEAP1 gene in two different tissues.

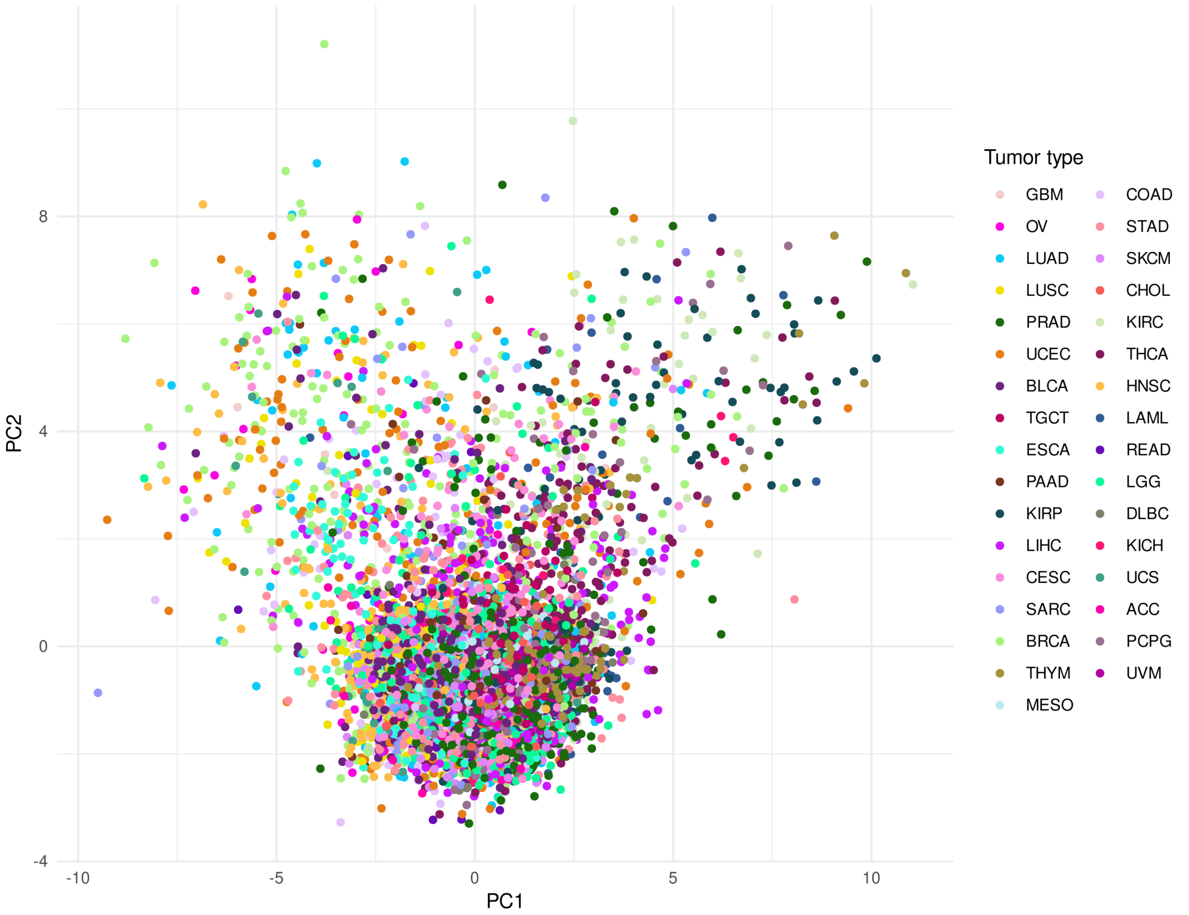

Supplementary Figure 7: Principal Component Analysis (PCA) of the 24 significant PSSs. The first two principal components are represented, points are colored by tumor type.

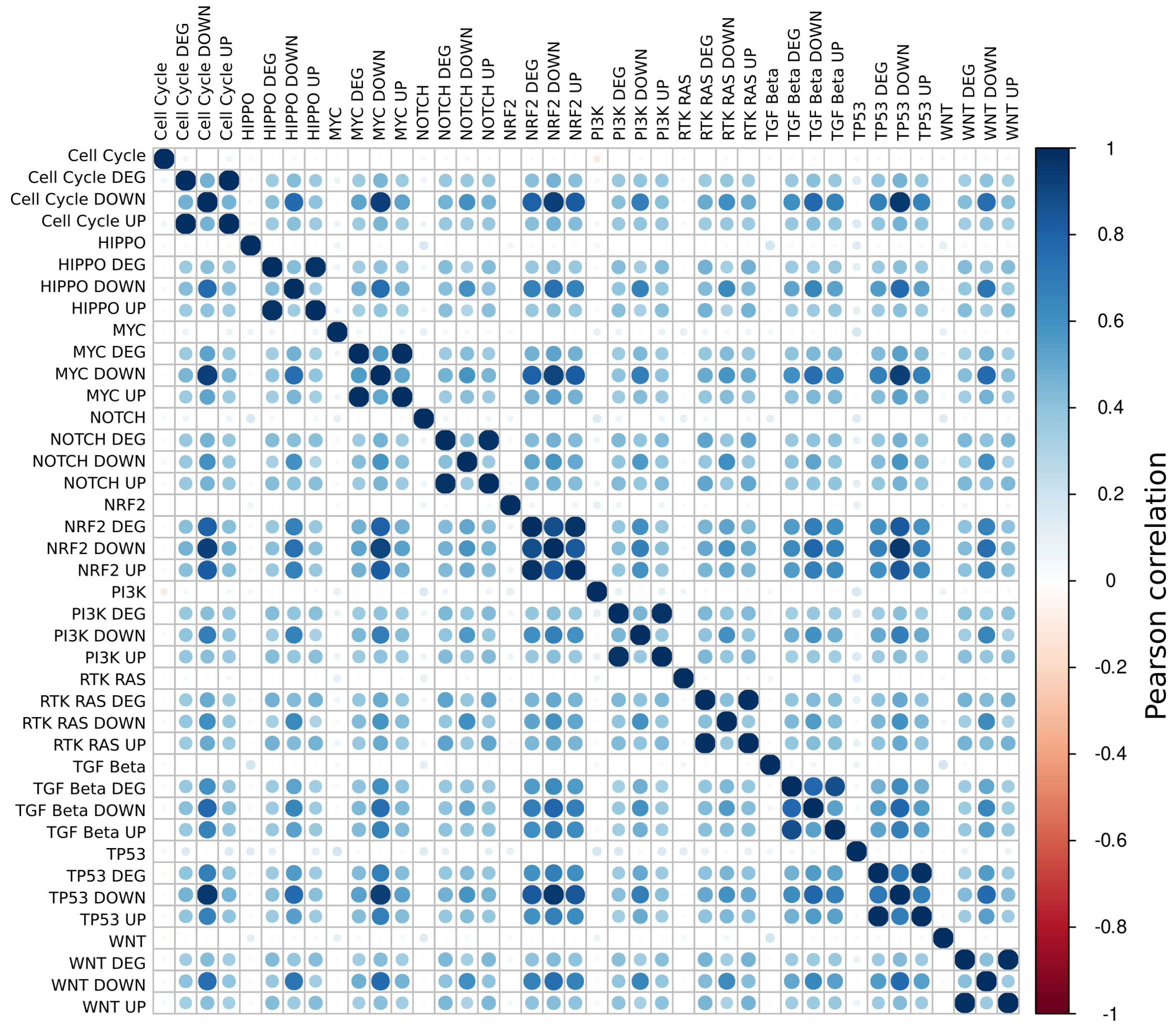

Supplementary Figure 8: Traits correlation analysis. Heatmap showing the correlations between all trait' pairs.

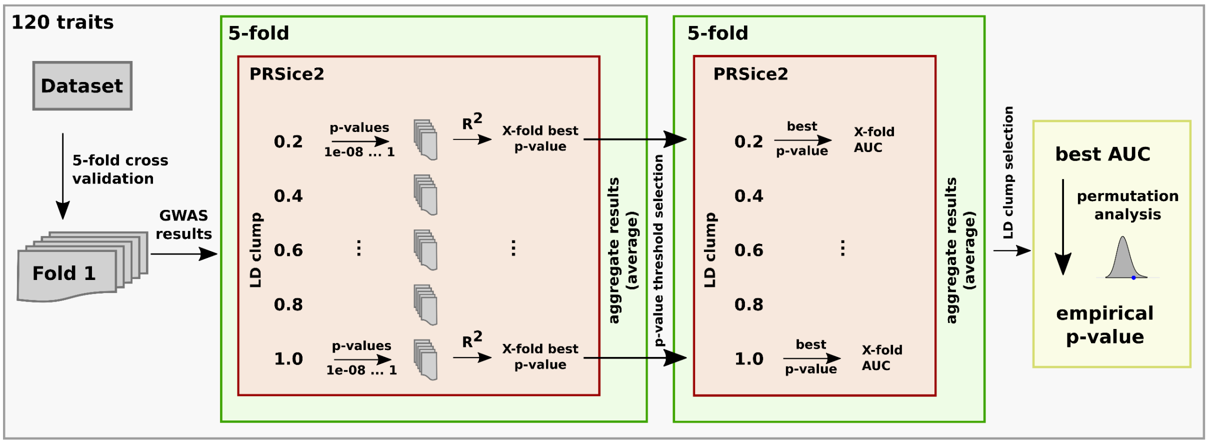

Supplementary Figure 9: PSS computational workflow. The workflow explains how PSSs are built and how their statistical significance is computed. For each trait, a 5-fold cross-validation is used to compute GWAS statistics. The best p-value thresholds across different LD clumps and averaged across the five folds are computed using PRSice-2. Then, the AUC performance scores are computed for each LD clump at the corresponding best p-value threshold. The best performing combination of p-value threshold and LD clump are used to generate each trait's PSS. Finally, a permutation approach is used to compute empirical p-values for each trait comparing each observed AUC value and the corresponding AUC baseline reference distribution.
